# Combination of On-Line and Off-Line Two-Dimensional Liquid Chromatography-Mass Spectrometry for Comprehensive Characterization of mAb Charge Variants and Precise Instructions for Rapid Process Development

**DOI:** 10.3390/ijms242015184

**Published:** 2023-10-14

**Authors:** Xiaoqing Jin, Bingfang He

**Affiliations:** 1 College of Biotechnology and Pharmaceutical Engineering, Nanjing Tech University, Nanjing 211816, China; jxq19810905@163.com; 2 School of Pharmaceutical Sciences, Nanjing Tech University, Nanjing 211816, China

**Keywords:** monoclonal antibody, charge variants, heterogeneity, characterization, 2D-LC-MS, on-line, off-line, precision process development

## Abstract

Charge variants, as an important quality attribute of mAbs, must be comprehensively characterized and monitored during development. However, due to their complex structure, the characterization of charge variants is challenging, labor-intensive, and time-consuming when using traditional approaches. This work combines on-line and off-line 2D-LC-MS to comprehensively characterize mAb charge variants and quickly offer precise instructions for process development. Six charge variant peaks of mAb 1 were identified using the developed platform. Off-line 2D-LC-MS analysis at the peptide level showed that the acidic peak P1 and the basic peaks P4 and P5 were caused by the deamidation of asparagine, the oxidation of methionine, and incomplete C-terminal K loss, respectively. On-line 2D-LC-MS at the intact protein level was used to identify the root causes, and it was found that the acidic peak P2 and the basic peak P6 were due to the glutathionylation of cysteine and succinimidation of aspartic acid, respectively, which were not found in off-line 2D-LC-MS because of the loss occurring during pre-treatment. These results suggest that process development could focus on cell culture for adjustment of glutathionylation. In this paper, we propose the concept of precision process development based on on-line 2D-LC-MS, which could quickly offer useful data with only 0.6 mg mAb within 6 h for precise instructions for process development. Overall, the combination of on-line and off-line 2D-LC-MS can characterize mAb charge variants more comprehensively, precisely, and quickly than other approaches. This is a very effective platform with routine operations that provides precise instructions for process development within hours, and will help to accelerate the development of innovative therapeutics.

## 1. Introduction

Biopharmaceuticals are the fastest growing sector of the pharmaceutical industry. Of the top 100 best-selling drugs in 2022, six of the top ten were biotherapeutics, including two mRNA vaccines and four antibody-based proteins (three mAbs and one Fc fusion protein) [1]. More than 100 antibody-based biotherapeutics have been approved in different countries all around the world, and approximately 140 antibody-based therapeutics are undergoing late-stage evaluation for approval to market [2]. The global antibody market is projected to exhibit an annual growth rate of approximately 15% in the next decade [3,4,5]. One significant difference between mAbs and conventional small molecule drugs is the much higher molecular weight and structural complexity of the mAb molecule, especially its heterogeneity in size, charge, and glycoforms. As one of the most important quality attributes of mAbs, charge heterogeneity can influence its stability, immunogenicity, potency, pharmacodynamics, and pharmacokinetics [6,7]. The charge heterogeneity of mAbs is typically the consequence of post-translational modifications (PTMs) that can occur in cell culture, purification and storage, and should be monitored for consistency in quality, safety, and efficiency.

A typical mAb charge profile contains one major and several minor species, defined as acidic, main, and basic species. Species with relatively lower isoelectric points (pIs) than the major species are defined as acidic species, while species with relatively higher pIs are defined as basic species [8]. Acidic variants typically arise from deamidation [9], sialylation [10], and glycation [11]. Basic variants are typically caused by incomplete C-terminal lysine truncation [12], C-terminal amidation [13], and isomerization of aspartate residues [14]. Cation exchange chromatography (CEX) is the classical method for evaluating charge variants of mAbs and is widely used in release testing and stability studies [15]. However, CEX offers only limited information, such as the relative ratio of different peaks, and it has a limited ability to explain the cause of different charge variants.

Liquid chromatography-mass spectrometry (LC-MS), which usually refers specifically to one-dimensional liquid chromatography tandem mass spectrometry, is the most popular tool for the structure characterization of mAbs and can offer detailed structure information of charge variants. It is labor-intensive and time-consuming to perform peak collection and the subsequent characterization, and it also may introduce pre-treatment-induced artifact data [16,17]. To improve the efficacy and capacity of LC-MS for characterization, 2D-LC-MS has been developed in recent decades. The additional dimension opens up a wide range of possibilities for system expansion and specific applications [18]. 2D-LC-MS can fractionate and characterize peaks of interest, and is well suited for the extended characterization of mAbs. Bathke et al. developed a 2D-LC (IEC × RP)-MS method to characterize mAbs in a fully automated process using online peak fractionation, reduction, and subsequent MS analysis [17]. However, the on-line reduction step rendered some reversible modifications undetectable, such as glutathionylation.

The biopharmaceutical industry is growing rapidly, and many companies are allocating more resources to develop innovative therapeutics and biosimilars. The increasingly fierce competition is pushing developers to accelerate the speed of development and launch to the market. While the traditional practice for process development is based on a trial-and-error approach, without targeted optimization, many experiments are usually required for a robust process, which is time-consuming, labor-intensive, costly, and inefficient. Therefore, there is an urgent need to transform process development, which is the core of CMC (chemistry, manufacture, control). In this paper, we propose the concept of precision process development (PPD) in the biopharmaceutical industry. PPD aims to transform process development from a traditional trial-and-error approach to precise and targeted development. Based on comprehensive and timely data, PPD can accelerate process development through targeted optimization and this can significantly increase efficiency and reduce the cost and time of development. The critical point is analytical methods that can quickly offer real and comprehensive data.

In this study, we demonstrated that a combination of on-line and off-line 2D-LC-MS comprehensively characterized the charge variants of mAbs using routine operations, and quickly offered more valuable information to better understand the mAbs in process development. The glutathionylation of cysteine obtained from the on-line 2D-LC-MS quickly and precisely guided the cell culture process development. By combining on-line and off-line 2D-LC-MS, the developed platform was very effective for precision process development to accelerate the development of mAbs.

## 2. Results

### 2.1. Charge Variant Profile of mAb 1 by CEX Liquid Chromatography

**Figure 1:**
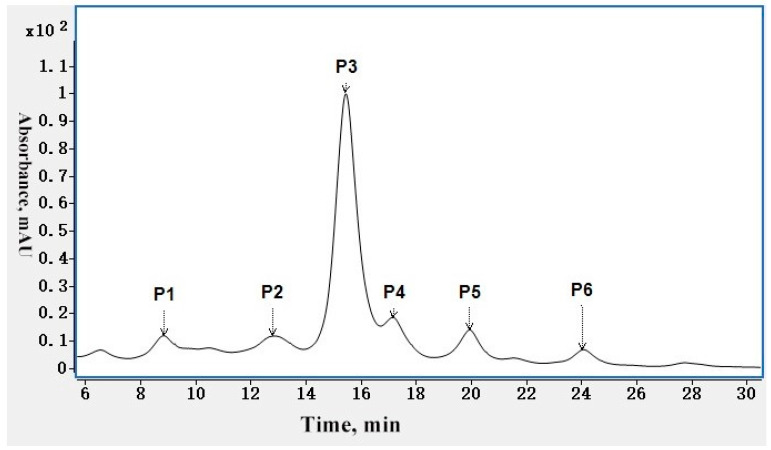
CEX charge variants profile of mAb 1. P1 and P2 are acidic peaks, P3 is main peak, and P4, P5 and P6 are basic peaks.

CEX separates charge variants primarily based on the surface charge of solvent-exposed residues combined with hydrophobic interactions [19]. Typically, mAbs have one main peak, which is the greatest proportion of the charge variants, several acidic peaks that elute before the main peak, and several basic peaks that elute after the main peak. CEX can provide the proportions of different charge variants, which is very useful for monitoring the consistency among different batches and the trends in stability studies. Figure 1 shows the charge variant profile of mAb 1 by CEX. There is one main peak denoted as P3, two acidic peaks denoted as P1 and P2, and three basic peaks denoted as P4, P5, and P6 in the figure. The ratios of the main peak, acidic peaks, and basic peaks were 12.70%, 71.49%, and 15.81%, respectively. More details are listed in Supplementary Materials Table S1 and Figure S2.

### 2.2. Characterization of Charge Variants at the Peptide Level by Off-Line 2D-LC-MS

**Figure 2:**
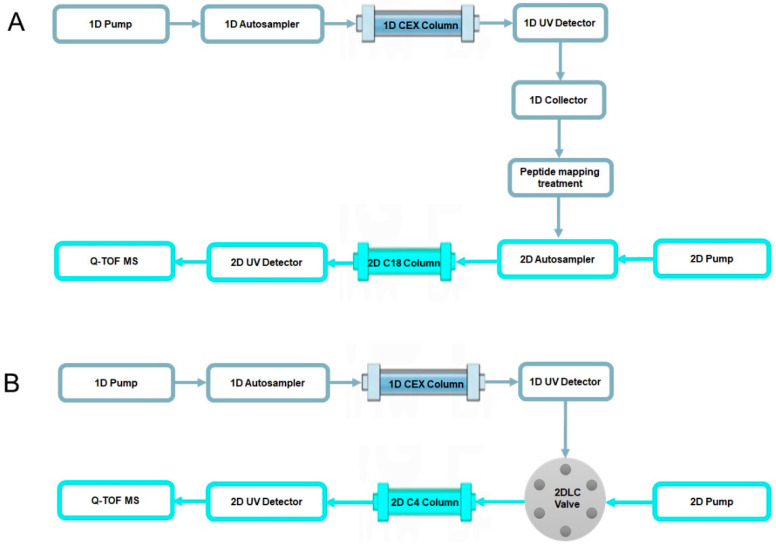
Scheme of off-line 2D-LC-MS (**A**) and on-line 2D-LC-MS (**B**).

**Figure 3:**
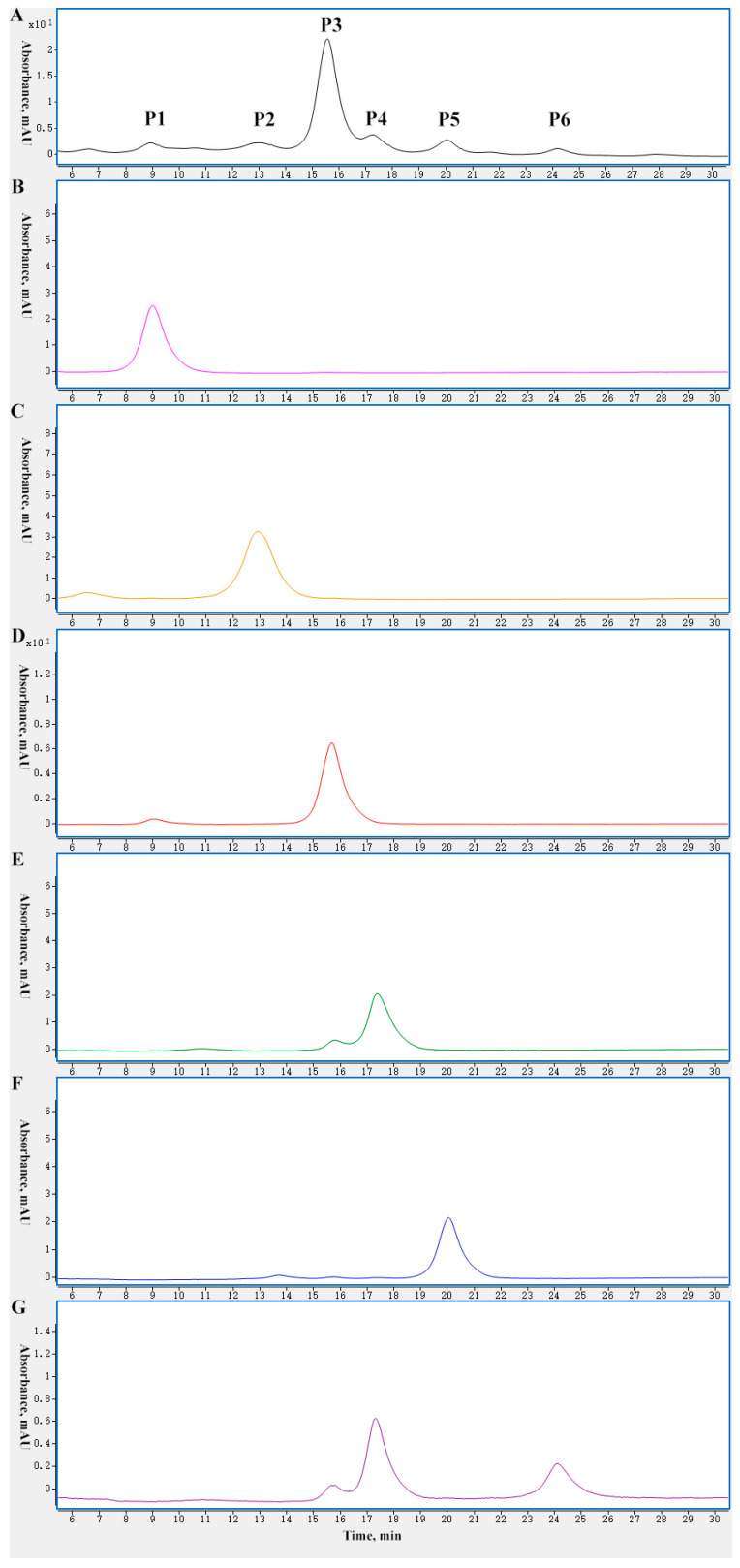
Chromatograms of six charge variants after fractionation collection of mAb 1 by off-line 2D-LC-MS. (**A**) CEX chromatogram of mAb 1, P1 and P2 are the acidic peaks, P3 is the main peak, P4, P5 and P6 are the basic peaks. (**B**) CEX chromatogram of P1 fraction after collection. (**C**) CEX chromatogram of P2 fraction after collection. (**D**) CEX chromatogram of P3 fraction after collection. (**E**) CEX chromatogram of P4 fraction after collection. P4 fraction was not completely separated with P3 because of the adjacent retention time. (**F**) CEX chromatogram of P5 fraction after collection. (**G**) CEX chromatogram of P6 fraction after collection. P6 fraction showed P3 and P4 peak besides P6 peak in CEX chromatogram because P6 changed in the collection and concentration step.

Peptide mapping is a common and effective method for testing modifications of mAbs. An off-line 2D-LC-MS system, as shown in Figure 2A, was used to characterize the six charge variants at the peptide level. The six charge variants were fractionated according to their eluting times in 1D-LC (CEX). Before the peptide level analysis, the six peak fractions were analyzed by CEX to confirm the fractionation efficiency, and the chromatograms are shown in Figure 3. The P1, P2, P3, and P5 fractions showed only one peak with a consistent retention time, which indicated that the CEX peak fraction was effective. For the P4 fraction, the chromatogram showed P4 was a major peak and P3 was a minor peak, which was due to incomplete separation because of the closely adjacent retention times of P3 and P4. It was interesting that the chromatogram of the P6 fraction showed a low P6 peak with a high P4 peak and low P3 peak. The P3 peak and P4 peak were far from P6 in the CEX chromatogram. Hence, it was impossible for the tailing of the P3 and P4 peaks to cause this phenomenon.

Table 1 summarizes the modifications of six charge variants of mAb 1 by off-line 2D-LC-MS at the peptide level. The protein sequence coverages are listed in Supplementary Materials Table S3. The masses of identified peptides digested from each peak collection, including peptides with modifications, are listed in Supplementary Materials Table S4 to Table S9. P3 was the main peak with a 99.93% C-terminal K loss in the heavy chain, 5.48% deamidation of N387 in the heavy chain, 5.37% deamidation of N30 in the light chain, 4.51% oxidation of M255 in the heavy chain, and 0.66% oxidation of M431 in the heavy chain, all of which are common PTMs for mAbs. The acidic peak P1 showed a comparable C-terminal K loss as the main peak P3, and with much higher deamidation of N387 (98.41%) and N30 (34.86%), slight lower oxidation of M255, and slightly higher oxidation of M431 than the main peak P3. Therefore, the acidic peak P1 was mainly due to the deamidation of N387 and N30. This was in good agreement with other studies [20,21]. The PTMs of the acidic peak P2 were comparable to those of the main peak P3, and the use of off-line data alone could not offer the exact reasons for the acidic peak P2. The basic peak P4 was very close to the main peak P3 in the CEX chromatogram, and showed higher deamidation of N387, lower deamidation of N30, a lower C-terminal K loss, and higher oxidation of M255 and M431. Lower C-terminal K loss and higher oxidation of M255 and M431 were the main reasons for the basic peak P4. Compared with the main peak P3, the basic peak P5 showed a much lower C-terminal K loss (48.56% vs. 99.93%), and the incomplete C-terminal K loss was the main cause of the basic peak P5. The higher oxidation of M255 and M431 also contributed to the basic peak P5. The basic peak P6 showed a lower C-terminal K loss and higher oxidation of M255 and M431 than the main peak P3. However, the basic peak P6 was more basic than the basic peak P5 in the CEX chromatogram, but showed a lower C-terminal K loss and less oxidation of methionine. Therefore, the C-terminal K loss and oxidation of methionine was not the cause of the basic peak P6. To find the root cause, the molecular weight of the collected P6 fraction was confirmed and the details are described later. 

In summary, the acidic peak P1 and the basic peaks P4 and P5 were caused by the deamidation of asparagine, the oxidation of methionine, and the incomplete C-terminal K loss, respectively, while the root cause of the acidic peak P2 and the basic peak P6 were not identified by off-line 2D-LC-MS at the peptide level. The corresponding modifications of the CEX peak root cause were validated by MS/MS at the peptide level. The details of the MS/MS spectra and fragment ions are in Supplementary Materials Figures S4–S13 and Tables S10–S19.

### 2.3. Characterization of Charge Variants at the Intact Protein Level by on-Line 2D-LC-MS

**Figure 4:**
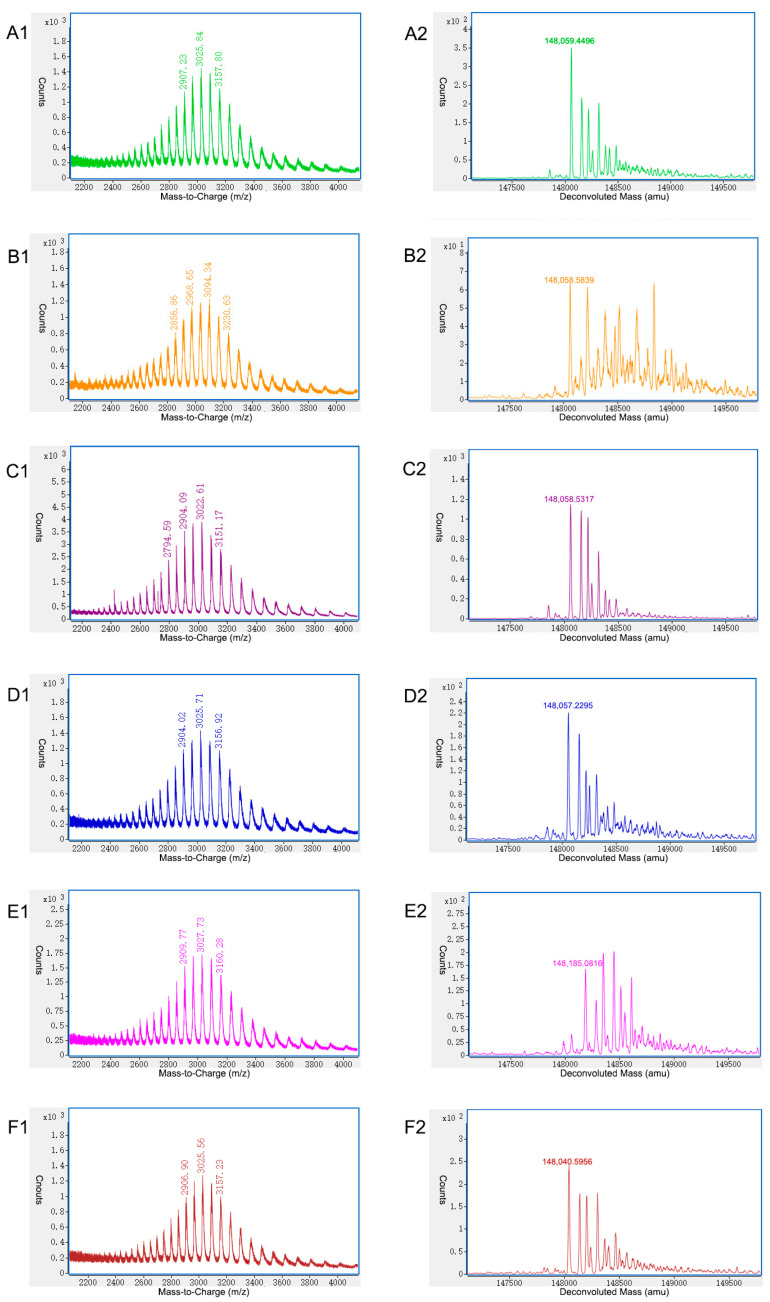
Raw (**left**) and deconvoluted (**right**) MS spectra of charge variants of mAb 1 by on-line 2D-LC-MS. (**A**) Raw (**left**) and deconvoluted (**right**) MS spectra of P1. (**B**) Raw (**left**) and deconvoluted (**right**) MS spectra of P2. (**C**) Raw (**left**) and deconvoluted (right) MS spectra of P3. (**D**) Raw (**left**) and deconvoluted (right) MS spectra of P4. (**E**) Raw (**left**) and deconvoluted (**right**) MS spectra of P5. (**F**) Raw (**left**) and deconvoluted (**right**) MS spectra of P6.

On-line 2D-LC-MS was used to quickly characterize the charge variants without pre-treatment to reduce potential method-induced artifacts. An on-line 2D-LC-MS system with CEX as 1D-LC and RP-Polyphenyl as 2D-LC Q-TOF-MS, as shown in Figure 2B, was used to characterize the six charge variants at the intact protein level. Raw and deconvoluted MS spectra of the charge variants of mAb 1 by on-line 2D-LC-MS are shown in Figure 4.

**Figure 5:**
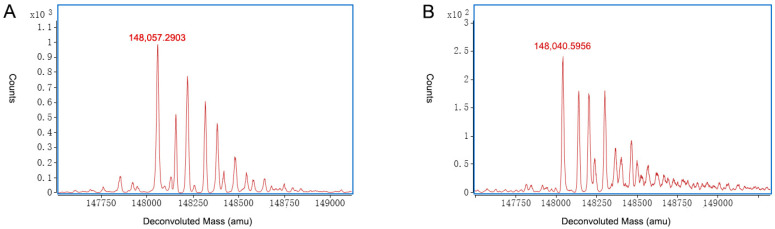
Comparison of deconvoluted MS spectra of the basic peak P6 from off-line and on-line 2D-LC-MS. (**A**) Data in molecular weight confirmation test from off-line 2D-LC-MS method. (**B**) Data from on-line 2D-LC-MS method.

P3 was the main peak, and it had a detected molecular weight of 148,058.5317 Da, which was confirmed to be a variant with the modifications 2 G0F + 2 K loss. The mass error was only −2.0323 ppm compared with the theoretical mass of 148,058.8326 Da. The mass accuracy of the molecular weight of the main peak was checked with triplicates. The details are listed in Supplementary Materials Table S2 and Figure S3. Two other variants in the main peak P3 were identified as G0F/G1F + 2 K loss, and G0F/G2F (or 2 G1F) + 2 K loss. Glycans such as G0F, G1F, and G2F are very common in mAbs and are usually the predominant glycans [22]. A C-terminal K loss is also one of the most common modifications and should be considered as low risk because it has no effect on mAb structure, stability, or potency [23]. The acidic peak P1 showed nearly the same modifications as the main peak P3. By combining the data from off-line 2D-LC-MS, high-level deamidation of asparagine was found to be the main cause of the acidic peak P1. The acidic peak P2 had similar modifications to the main peak P3, such as a 2 K loss and common glycans, including G0F, G1F, and G2F. Additionally, the acidic peak P2 had a unique modification-glutathionylation of cysteine that was not found by off-line 2D-LC-MS at the peptide level. Glutathionylations are expected in the acidic regions because glutathione adds two carboxylic acid groups and cysteine contributes one [11]. One MS species of the acidic peak P2 of 148,673.7761 Da was identified as a molecule with 2 G0F + 2 K loss + 2 glutathionylations indicated by a mass shift of +610 Da that matched two glutathionylations of cysteines. The other two MS species of the acidic peak P2 were identified as molecules with G0F/G1F + 2 K loss + 2 glutathionylations, and G0F/G2F (or 2 G1F) + 2 K loss + 2 glutathionylations with mass errors of 8.3638 ppm and 8.2165 ppm, respectively. The basic peak P4 showed nearly the same modifications as the main peak P3. As shown in the results at the peptide level by the off-line 2D-LC-MS method, the basic peak P4 was mainly due to high levels of oxidation of methionine. The three major MS species of the basic peak P5 had a mass shift of +128 compared with the main peak P3, and was identified as 2 G0F + 1 K loss, G0F/G1F+ 1 K loss, and G0F/G2F (or 2 G1F) + 1 K loss, with mass errors of −12.4572 ppm, 0.4294 ppm, and −7.5079, respectively. An incomplete K loss was one of the main reasons for the basic mAb variants [13,24], and the basic peak P5 was due to an incomplete K loss compared with the main peak P3. The reason for the basic peak P5 was the same as that determined by off-line 2D-LC-MS. The basic peak P6 was due to succinimidation because of a mass shift of −18 Da that matched the succinimide of an aspartic acid variant from the main peak P3. The three main MS species of P6 with molecular weights of 148,040.5956 Da, 148,202.2814 Da and 148,365.6753 Da were identified as succinimidation of the variants 2 G0F + 2 K loss, G0F/G1F + 2 K loss, and G0F/G2F (or 2 G1F), respectively, and the mass errors were −8.1767 ppm, −11.2548 ppm, and −2.8133 ppm, respectively. According to the off-line 2D-LC-MS analysis at the peptide level, no succinimidation was found. Therefore, the basic peak P6 fraction was analyzed to confirm the molecular weight and compared with the on-line 2D-LC-MS data, as shown in Figure 5. Figure 5A shows the result of the molecular weight test, which indicated that the peak with the detected molecular weight of 148,057.2903 Da was confirmed to be the variant with modifications 2 G0F + 2 K loss, and the mass error was −10.4168 ppm, compared with the theoretical mass of 148,058.8326 Da. Figure 5B shows the on-line 2D-LC-MS data, which indicated that the peak with the detected molecular weight of 148,040.5956 Da was confirmed to be the variant with modifications 2 G0F + 2 K loss + succinimide, and the mass error was −8.1767 ppm compared with the theoretical mass of 148,041.8061 Da. Briefly, the on-line 2D-LC-MS data illustrated that the basic peak P6 included variants with succinimidation, while no succinimidation was found in the off-line 2D-LC-MS or molecular weight confirmation tests. It was speculated that the succinimidation form of aspartic acid in the basic peak P6 had transferred to aspartic acid in the main peak P3 and isoaspartic acid in the basic peak P4 during the collection and concentration step.

In summary, according to the on-line 2D-LC-MS data at the intact protein level, the basic peak P5 was due to the incomplete C-terminal K loss, the acidic peak P2 was due to the glutathionylation of cysteine, and the basic peak P6 was due to the succinimidation of aspartic acid.

## 3. Discussion

MAbs are large molecules with highly complex structures, and their variants are caused by different modifications. There is no one single method that can provide complete structure information of mAbs, and therefore different orthogonal analytical methods are required to analyze mAbs for their development [25]. A combination of on-line and off-line 2D-LC-MS offers more modification information on the charge variants of mAb 1 and finds the root cause of charge variants that can be utilized for precision process development to increase efficiency and reduce the cost and time of development.

According to the on-line 2D-LC-MS analysis, it was determined that the main peak P3 was mainly composed of variants with a 2 K loss and different glycans, such as 2 G0F, G0F/G1F, and G0F/G2F (or 2 G1F), which are very common in the modifications of mAbs. Off-line 2D-LC-MS offered more detailed modifications, including a nearly complete C-terminal K loss, some deamidation of asparagine, and oxidation of methionine. For the acidic peak P1, the main modification was a very high level of deamidation of N387 and N30, which was identified by the off-line 2D-LC-MS method, while on-line 2D-LC-MS could not offer such precise useful information at the intact protein level. Because the deamidation had only a +0.9840 mass shift, these subtle mass changes were very challenging to distinguish at the intact protein level [26]. The on-line 2D-LC-MS data of the acidic peak P2 illustrated glutathionylation of cysteine from the intact protein level, and this was the main reason for the acidic peak P2, which was reported previously [11]. However, no glutathionylation was found by off-line 2D-LC-MS, and fewer C-terminal K losses, similar levels of deamidation of asparagine, and higher levels of oxidation of methionine were found. This was because protein glutathionylation is a reversible process [27,28,29] and is reduced in the pre-treatment step of reduction by DTT. For the basic peak P4, no exact modifications were obtained by the on-line 2D-LC-MS method, while off-line 2D-LC-MS provided detailed modifications, such as higher oxidation of methionine, higher deamidation of asparagine, and fewer C-terminal K losses than in the main peak P3. Oxidation of methionine residues causes a shift to the basic side on WCX-10 chromatography. Compared to the side chain of methionine residues, methionine sulfoxide, which is the stable product of methionine oxidation, is more basic [30]. The basic species caused by oxidation of methionine have been reported by several studies [10,31,32,33]. Oxidation and fewer C-terminal K losses were the main reason for the basic peak P4. The basic peak P5 showed fewer truncations of C-terminal K than the main peak P3 from the on-line 2D-LC-MS method, which was confirmed by the off-line 2D-LC-MS method. The off-line 2D-LC-MS method also showed more oxidation of methionine and higher deamidation of asparagine than the main peak P3. C-terminal K losses are an important reason for charge variants, in which a higher C-terminal K loss than in the main peak causes acidic variants, and fewer C-terminal K losses than in the main peak cause basic variants. The incomplete C-terminal K loss and higher oxidation of methionine were the main cause for the basic peak P5. The basic peak P6 showed succinimidation of aspartic acid from on-line 2D-LC-MS data, less C-terminal K loss, slightly higher oxidation of methionine, and similar deamidation of asparagine compared to the main peak P3 from off-line 2D-LC-MS data. An interesting phenomenon was that the basic peak P6 fraction showed a low P6 peak with a high P4 peak and a low P3 peak in a conformation test by CEX. The molecular weight confirmation test of the fraction of the basic peak P6 showed no succinimidation, which was different from the on-line 2D-LC-MS results. Additionally, the off-line 2D-LC-MS data also showed no succinimidation for the fraction of the basic peak P6. Succinimide-mediated reactions of aspartic acid in proteins have been extensively studied in the past [10,31,32,34,35,36,37]. Succinimide, as an aspartate isomerization intermediate, has been confirmed experimentally to contribute to the formation of basic species because of the loss of a negative charge compared to aspartate [35,37,38]. The on-line 2D-LC-MS data demonstrated the succinimidation of the basic peak P6, because the accurately measured mass loss was in accordance with the theoretical mass loss during succinimide formation (−18 Da). In contrast, the confirmation test of the molecular weight of the basic peak P6 and off-line 2D-LC-MS method at the peptide level failed to detect succinimide. Succinimide is prone to spontaneous hydrolysis to isoaspartate and aspartate, and therefore we speculated that succinimide converted to isoaspartate and aspartate during the collection and concentration step. This was similar to a study by Grace that showed that succinimide was hydrolyzed to isoaspartate and aspartate in less than 2 h in the process of reduction and alkylation under denaturing conditions [37].

In summary, six charge variant peaks of mAb 1 were comprehensively characterized. It should be emphasized that the on-line 2D-LC-MS offered the exact modifications of two charge variants while off-line 2D-LC-MS did not. The acidic peak P2 was mainly caused by the glutathionylation of cysteine at the intact protein level, which was not found at the peptide level because of the reduction by DTT during pre-treatment. The basic peak P6 showed the succinimidation of aspartic acid from on-line 2D-LC-MS, a low P6 peak with a high P4 peak and a low P3 peak in a conformation test by CEX, and no succinimidation in the molecular weight confirmation test or off-line 2D-LC-MS, which was due to succinimide changing to isoaspartate and aspartate during the collection and concentration step.

Recently, multi-dimensional liquid chromatography-tandem mass spectrometry (mD-LC-MS) has been developed for on-line and automatic characterization. Gstöttner et al. developed a 4D-LC-MS system implementing CEX as one dimension, reduction as the second dimension, trypsin digestion as the third dimension, and peptide mapping as the fourth dimension for the automated separation of mAbs, fractionation of peaks, reduction, tryptic digestion, and reversed-phase (RP) separation of the resulting peptides followed by MS detection. The entire separation and analytical process for an unknown peak was performed in less than 1.5 h, leading to a significant time savings [39]. Additionally, an innovative 5D-LC-MS system incorporated in parallel trypsin and Lys C on-column digestion enabled the characterization of bispecific antibodies with excellent sequence coverage [18]. However, more is not always better. More dimensions mean more complexity in the system and the connection between dimensions becomes difficult to operate, which also suggests a high cost in capital expenditure. Furthermore, although the testing time of these mD-LC-MS methods is lower than traditional approaches, the time for the setup of these mD-LC-MS systems is usually very long. No matter what methods are used, the most important requirement is that the method quickly offers valuable information with simple operation and low cost. The developed 2D-LC-MS system utilized a commercialized Agilent 2D-LC and Q-TOF MS without a complex connection, which was very easy to operate at a low cost. For example, the on-line 2D-LC-MS method completed six charge variant characterizations from the intact protein level with only 0.6 mg of mAb within 6 h. The traditional preparative CEX chromatography for MS characterization typically requires more than 100 mg of protein and takes weeks [40]. Moreover, the on-line 2D-LC-MS method operates under physiological conditions without any pre-treatment, allowing for analysis in a native-like state, and reduces the artifacts from pre-treatment, such as DTT reduction in sample pre-treatment that generated a glutathionylation of cysteine loss in off-line 2D-LC-MS. Method-induced artifacts are common in sample pre-treatment, especially denaturing conditions for traditional peptide mapping protocols. For example, asparagine and methionine are highly susceptible to rapid modifications under typical digestion conditions (i.e., high pH, elevated temperatures, and long incubation times) [41]. Some labile modifications (e.g., succinimide) can also be lost during sample preparation [19]. It was important that the protocol minimized any artifacts that could confound the analysis of these modifications.

Our on-line 2D-LC-MS method quickly analyzed mAbs in a native-like state to find the root cause of charge variants without any potential artifacts. It made precise and targeted process development possible. Here, we propose the concept of precision process development (PPD) in the biopharmaceutical industry. Similar to precision medicine, which uses a person’s genetics, environment, and lifestyle to help determine the best approach to prevent or treat disease [42], precision process development utilizes real and comprehensive data from state-of-the-art technologies, including 2D-LC-MS and multi-omics, to help precisely guide process development. Different from traditional trial and error approaches, PPD emphasizes precise and targeted optimization that will significantly improve development efficiency with fewer experiments and less time and cost. The foundation of PPD is a comprehensive and timely data supply without any artifacts. For example, traditional approaches and off-line 2D-LC-MS could not find the root cause of the acidic peak P2 because of the loss of glutathionylation of cysteine during pre-treatment; therefore, it is impossible to solve the problem simply using a trial-and-error approach without the exact root cause. Our on-line 2D-LC-MS method illustrated that the root cause of the acidic peak P2 was the glutathionylation of cysteine. This guided us to reduce the ratio of the acidic peak P2 by adjusting glutathionylation of cysteine in cell culture. By combining recent progress in cell culture, cysteine and glutathione were shown to have crucial roles in maintaining the redox balance, productivity, and quality [43,44,45]. Therefore, process development should focus on cell culture optimization, such as basal media components and feeding strategies, especially cysteine and glutathione controls. PPD has tremendous potential to accelerate the development of biopharmaceutics and will transform the biopharmaceutical industry in the future.

## 4. Materials and Methods

### 4.1. Reagents

Sodium chloride was purchased from Sigma-Aldrich (Seelze, Germany). Sodium phosphate monobasic dihydrate, di-sodium hydrogen phosphate, dithiothreitol (DTT), formic acid (FA, LC-MS grade), acetonitrile (ACN, LC-MS grade) and deionized water (LC-MS grade) were purchased from Sigma-Aldrich (Darmstadt, Germany). Iodoacetamide (IAM), guanidine hydrochloride and ammonium bicarbonate were purchased from Sigma-Aldrich (St. Louis, MO, USA). Trypsin was purchased from Promega (Fitchburg, WI, USA). Mab 1 was from our lab.

### 4.2. Instrument

The 2D-LC-MS system was composed of an Agilent 1290 UPLC and 6545XT AdvanceBio Q-TOF. All instrument modules were from the 1290 Infinity line from Agilent Technologies: first (1D) and second (2D) dimension binary pumps; autosampler, thermostated column compartments, ultraviolet (UV) detectors, and fraction collector. The interface valve connecting the two dimensions of the system was set up with 20 µL sample loops made from 10 cm lengths of 0.0200 i.d. PEEK tubing. The mass spectrometer 6545XT AdvanceBio Q-TOF was a time-of-flight mass spectrometer instrument equipped with an Agilent JetStream electrospray ionization source.

The Agilent 1290 UPLC and 6545XT AdvanceBio Q-TOF were controlled by MassHunter software V10.0. Data were processed by Agilent MassHunter BioConfirm V10.0. The theoretical MW was confirmed by Sequence Manger V10.0.

### 4.3. CEX Liquid Chromatography Analysis

The experimental parameters of 1D CEX chromatography were identical in on-line 2D-LC-MS and off-line 2D-LC-MS. The charge variants of mAb 1 were separated by CEX using a ProPac^TM^ WCX-10 BioLC^TM^ analytical column (4 × 250 mm, 10 μm). One hundred micrograms of mAb 1 were injected each time and detected by the UV detector at 280 nm. The CEX mobile phase A (CEX-A) was 10 mM phosphate buffer (PB) at pH 7.5, and the CEX mobile phase B (CEX-B) contained 10 mM PB and 100 mM NaCl at pH 7.5. The gradient elution was increased from 0% CEX-B to 30% CEX-B in 38 min, with a 5 min equilibration using 100% CEX-A at the beginning and a 3 min regeneration using 100% CEX-B after gradient elution. The column temperature was set to 40 °C, and the flow rate was 0.5 mL/min.

### 4.4. Off-Line 2D-LC-MS Analysis

A scheme of the instrument used for off-line 2D-LC-MS is shown in Figure 2A. The 2D-LC-MS system with a CEX column as 1D and RP-C18 column as 2D were used for the charge variant characterization of mAb 1 at the peptide level.

The 1D CEX parameters were the same as those identified in the CEX liquid chromatography analysis. Six charge variants were collected according to their eluting time by multiple injections to obtain sufficient materials, and then were concentrated using a 10 kDa centrifugal tube filter. The purity of the collected fractions of the charge variants were confirmed by the same CEX liquid chromatography analysis, and the molecular weights were confirmed by the HPLC(RP- Polyphenyl)-MS method, the same as that of the 2D reversed-phase separations and MS in the on-line 2D-LC-MS system.

The antibody charge variants fractionated by 1D CEX were added to guanidine hydrochloride buffer, reduced by DTT, and alkylated with IAM. After the reduced and alkylated samples were desalted and the buffer was exchanged with 100 mM ammonium bicarbonate, the samples were digested with trypsin at 37 °C for 1 h. FA was added to stop the digestion. The peptides were injected onto a ZORBAX SB-C18 column (2.1 mm × 150 mm, 3.5 μm) coupled on-line to a Q-TOF MS for separation and detection. The column was operated at 45 °C with a flow rate of 0.3 mL/min. All fraction digests were analyzed with ultrapure water containing 0.1% FA as mobile phase A, and ACN containing 0.1% FA as mobile phase B. The gradient elution was increased from 2% mobile phase B to 35% mobile phase B in 57 min, with a 3 min equilibration using 98% mobile phase A at the beginning, a 15 min wash using 100% mobile phase B after the gradient elution, and a 2 min equilibration using 98% mobile phase A at the end. MS data were acquired in positive ion mode with a mass range of 250–3200 *m*/*z* at a rate of 3 Hz. MS/MS data were obtained simultaneously through the automatic MS/MS mode. The fragmentor voltage was set as 175V. The details of MS/MS parameters are in Supplementary Materials Figure S1.

### 4.5. On-Line 2D-LC-MS Analysis

A scheme of the instrument used for on-line 2D-LC-MS is shown in Figure 2B. The 2D-LC-MS system with a CEX column as 1D and an RP-Polyphenyl column as 2D was used for the charge variant characterization of mAb 1 at the intact protein level.

The 1D CEX parameters were the same as those mentioned in the CEX liquid chromatography analysis. Two-dimensional reversed-phase separations were performed using a BioResolve RP mAb polyphenyl column (2.1 mm × 50 mm, 2.7 µm). Mobile phase A was 0.1% (*v*/*v*) FA in water, and mobile phase B was ACN with 0.1% (*v*/*v*) FA. The gradient elution was increased from 5% mobile phase B to 95% mobile phase B in 3 min and maintained at 95% mobile phase B for 3 min, with a 5 min equilibration using 95% mobile phase A at the beginning and a 2 min equilibration using 95% mobile phase A after the gradient elution. The column temperature was 75 °C, and the flow rate 0.5 mL/min. The heart cutting mode was used for targeted fractions of different charge variants and then re-injected into the 2D column. Charge variants were detected at the intact protein level by Q-TOF MS. Data were acquired in positive mode with a mass range of 500–5000 *m*/*z* at a rate of 1 Hz. The fragmentor voltage was set as 380 V.

## 5. Conclusions

In conclusion, we combined on-line and off-line 2D-LC-MS for the comprehensive characterization of mAb 1 charge variants. Six charge variant peaks were identified at the intact protein level and peptide mapping level. The on-line 2D-LC-MS method that was very easy to operate with low cost identified the root cause of the charge variants quickly with only 0.6 mg of mAb within 6 h. Based on the concept of PPD that we proposed, the root cause of glutathionylation in the acidic peak P2 could guide targeted optimization in cell culture. The combination of on-line and off-line 2D-LC-MS, which is significantly different from a traditional LC-MS characterization workflow, can characterize the charge variants of mAbs more comprehensively, and effectively offers valuable information for the rapid development of biopharmaceutics.

## Figures and Tables

**Table 1:** Modification summary of six charge variants of mAb 1 by off-line 2D-LC-MS.

Modification	P1	P2	P3	P4	P5	P6
HC N387 Deamidation	98.41%	6.84%	5.48%	13.76%	9.08%	4.00%
LC N30 Deamidation	34.86%	4.08%	5.37%	2.63%	5.93%	7.02%
HC C-terminal K Loss	97.42%	95.19%	99.93%	94.62%	48.56%	93.13%
HC M255 Oxidation	2.61%	8.13%	4.51%	11.88%	8.70%	6.27%
HC M431 Oxidation	1.46%	1.32%	0.66%	3.61%	2.21%	1.66%

Note: HC means heavy chain, LC means light chain, N means Asparagine, M means Methionine.

## Data Availability

All data are contained within the article.

## Supplementary Materials

The following supporting information can be downloaded at: https://www.mdpi.com/article/10.3390/ijms242015184/s1.
