# Supplementary material for: Combination of On-Line and Off-Line Two-Dimensional Liquid Chromatography-Mass Spectrometry for Comprehensive Characterization of mAb Charge Variants and Precise Instructions for Rapid Process Development
Source: Int J Mol Sci. 2023 Oct 14;24(20):15184. doi: 10.3390/ijms242015184 (PMC10607358; doi:10.3390/ijms242015184)
Supplement: Supplementary file 1 [file ijms-24-15184-s001.zip › ijms-2607376-supplementary.pdf]

Supplementary Materials

Combination of On-Line and Off-Line Two-Dimensional Liquid Chromatography-Mass Spectrometry for Comprehensive Characterization of mAb Charge Variants and Precise Instructions for Rapid Process Development

Xiaoqing Jin, and Bingfang He

Table of content

Figure S1. Auto-MS/MS parameters for Off-line 2D-LC-MS……………………………………………Page S2

Figure S2. The overlay of CEX chromatogram in triplicate……………………………………………Page S3

Figure S3. The overlay of deconvoluted MS spectra in triplicate………………………………………Page S3

Figure S4. The MS/MS spectra of corresponding peptide with HC-N387 deamidation in P1………………Page S4

Figure S5. The MS/MS spectra of corresponding peptide without HC-N387 deamidation in P1……………Page S4

Figure S6. The MS/MS spectra of corresponding peptide with LC-N30 deamidation in P1…………………Page S4

Figure S7. The MS/MS spectra of corresponding peptide without LC-N30 deamidation in P1……………Page S4

Figure S8. The MS/MS spectra of corresponding peptide with HC-M255 oxidation in P4…………………Page S5

Figure S9. The MS/MS spectra of corresponding peptide without HC-M255 oxidation in P4………………Page S5

Figure S10. The MS/MS spectra of corresponding peptide with HC-M431 oxidation in P4………………Page S5

Figure S11. The MS/MS spectra of corresponding peptide without HC-M431 oxidation in P4……………Page S5

Figure S12. The MS/MS spectra of corresponding peptide with HC C-terminal K loss in P5………………Page S6

Figure S13. The MS/MS spectra of corresponding peptide without HC C-terminal K loss in P5……………Page S6

Table S1. The reproducibility of RT in CEX in triplicate……………………………………………………Page S6

Table S2. The reproducibility of mass accuracy of main peak in MS in triplicate…………………………Page S6

Table S3. The sequence coverage of each peak collection in off-line 2D-LC-MS…………………………Page S6

Table S4. The details of identified peptides digested from P1 in off-line 2D-LC-MS………………………Page S7

Table S5. The details of identified peptides digested from P2 in off-line 2D-LC-MS………………………Page S15

Table S6. The details of identified peptides digested from P3 in off-line 2D-LC-MS………………………Page S22

Table S7. The details of identified peptides digested from P4 in off-line 2D-LC-MS………………………Page S28

Table S8. The details of identified peptides digested from P5 in off-line 2D-LC-MS………………………Page S34

Table S9. The details of identified peptides digested from P6 in off-line 2D-LC-MS………………………Page S40

Table S10. The fragment ions of corresponding peptide with LC-N387 deamidation in P1………………Page S45

Table S11. The fragment ions of corresponding peptide without LC-N387 deamidation in P1……………Page S45

Table S12. The fragment ions of corresponding peptide with LC-N30 deamidation in P1…………………Page S45

Table S13. The fragment ions of corresponding peptide without LC-N30 deamidation in P1……………Page S46

Table S14. The fragment ions of corresponding peptide with HC-M255 oxidation in P4…………………Page S47

Table S15. The fragment ions of corresponding peptide without HC-M255 oxidation in P4………………Page S47

Table S16. The fragment ions of corresponding peptide with HC-M431 oxidation in P4…………………Page S48

Table S-17. The fragment ions of corresponding peptide without HC-M431 oxidation in P4………………Page S48

Table S18. The fragment ions of corresponding peptide with HC C-terminal K loss in P5………………Page S49

Table S19. The fragment ions of corresponding peptide without HC C-terminal K loss in P5……………Page S49

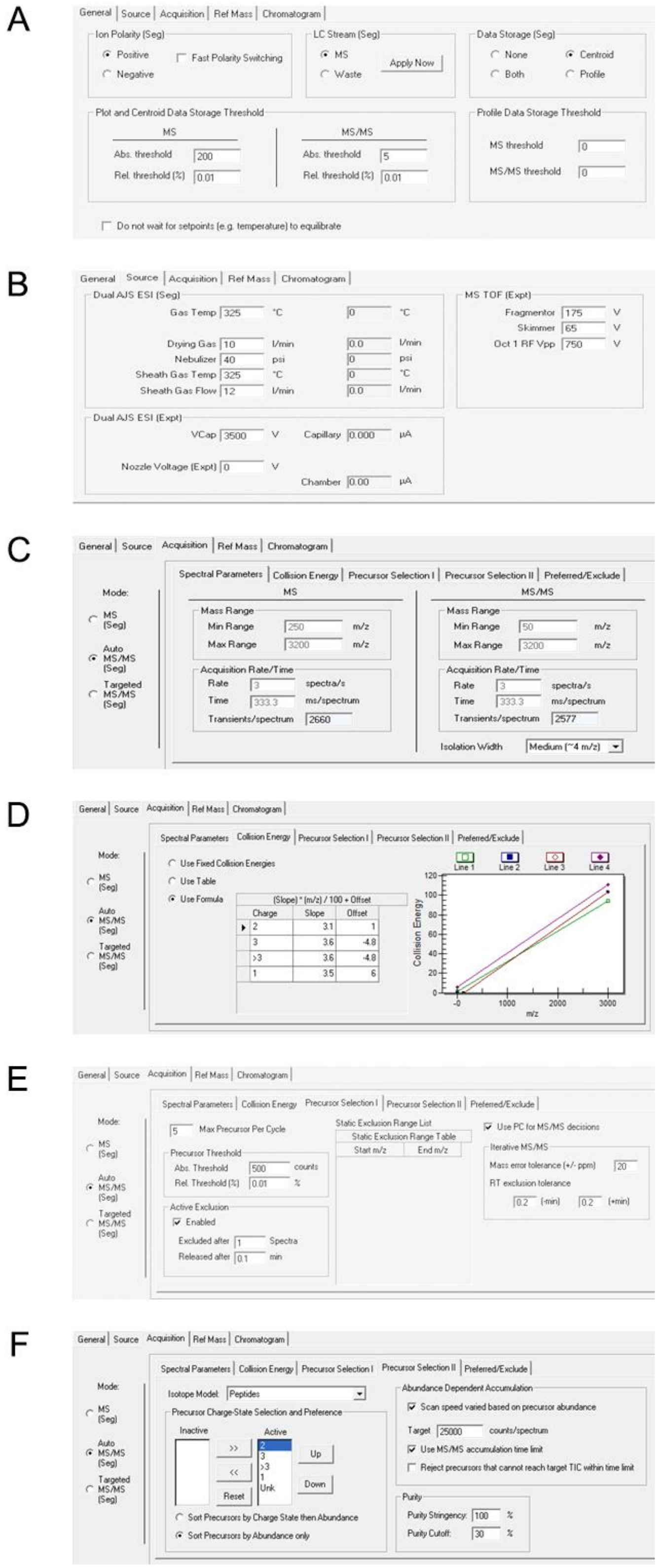

Figure S1 Auto-MS/MS parameters for Off-line 2D-LC-MS. A is general parameters, B is source parameters, C is acquisition-spectral parameters, D is acquisition-collision energy parameters, E is acquisition-precursor selection Ⅰ parameters, F is acquisition-precursor selection Ⅱ parameters.

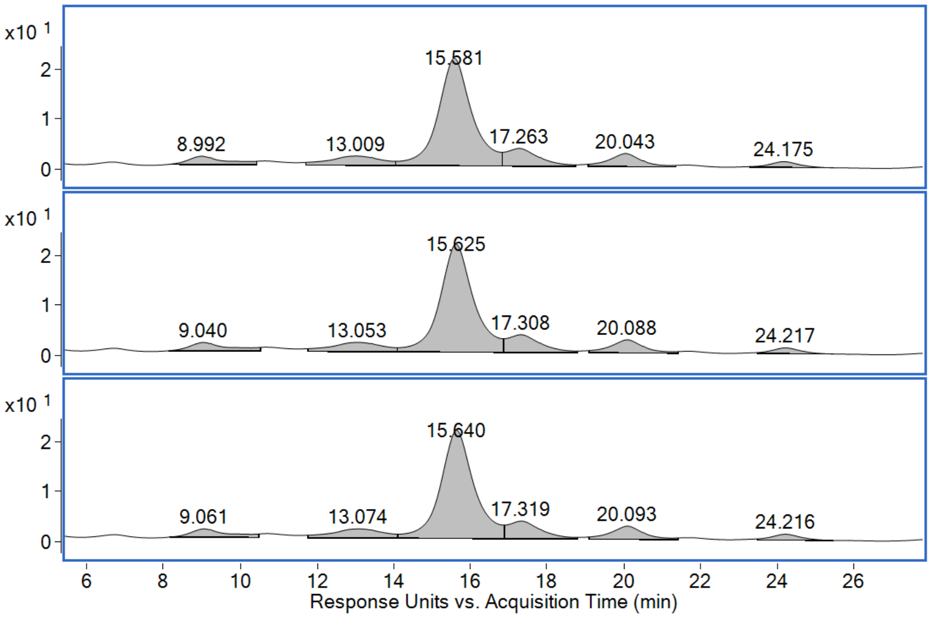

Figure S2 The overlay of CEX chromatogram in triplicate.

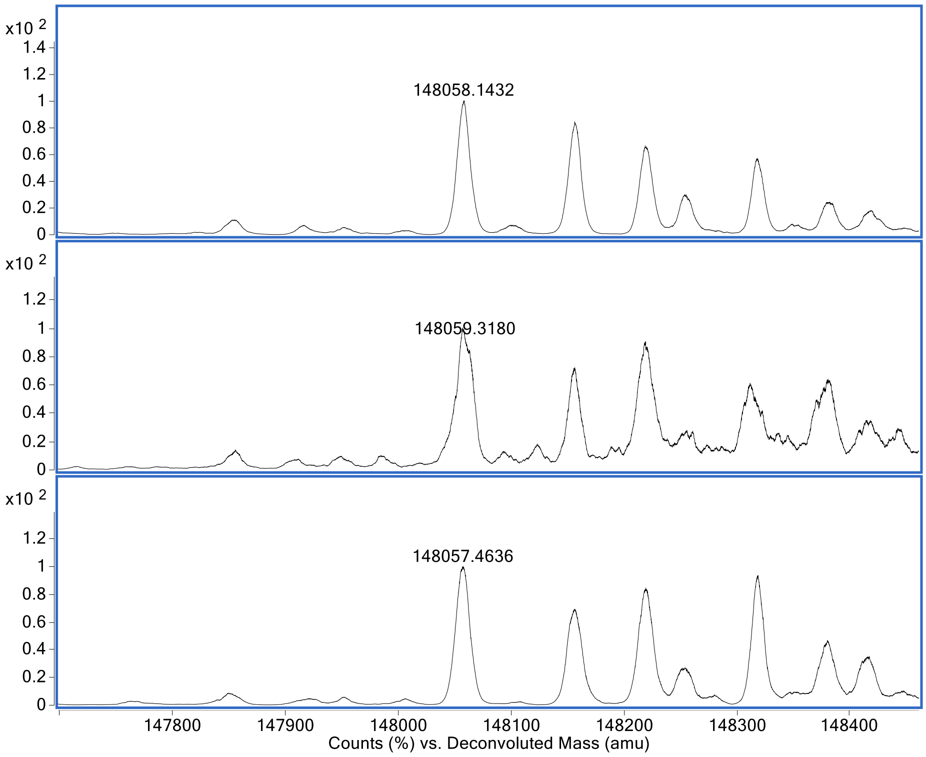

Figure S3 The overlay of deconvoluted MS spectra in triplicate.

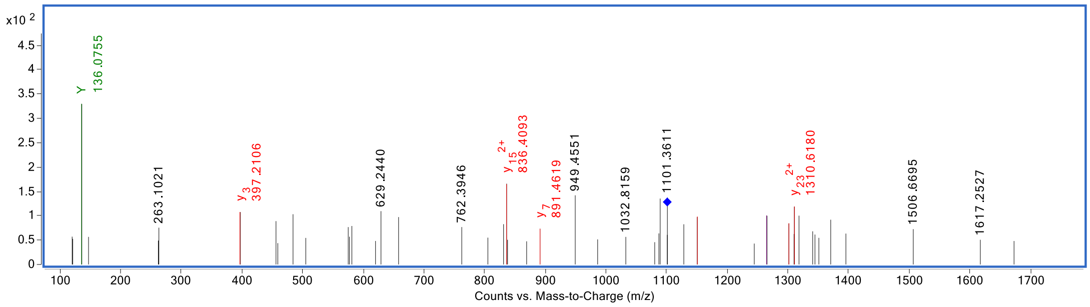

Figure S4 The MS/MS spectra of corresponding peptide with HC-N387 deamidation in P1.

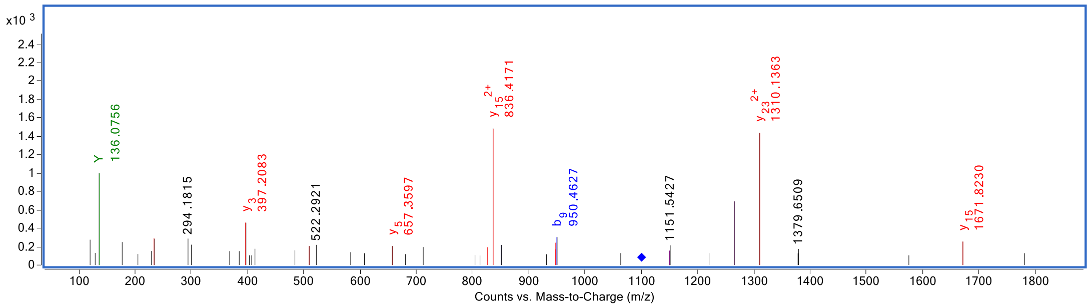

Figure S5 The MS/MS spectra of corresponding peptide without HC-N387 deamidation in P1.

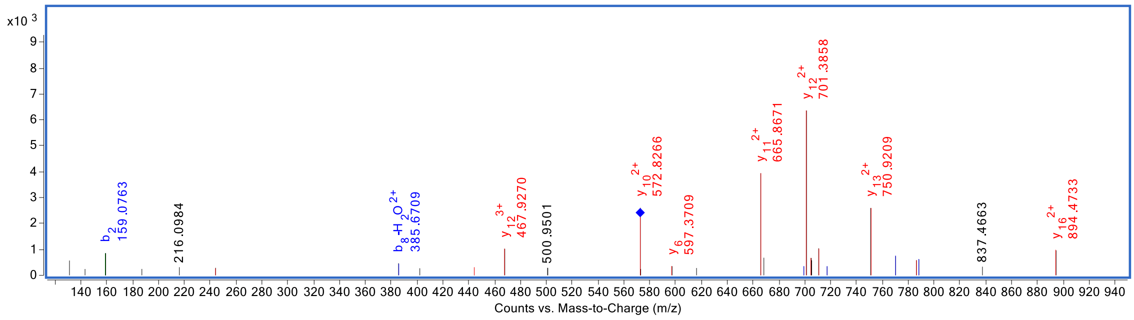

Figure S6 The MS/MS spectra of corresponding peptide with LC-N30 deamidation in P1.

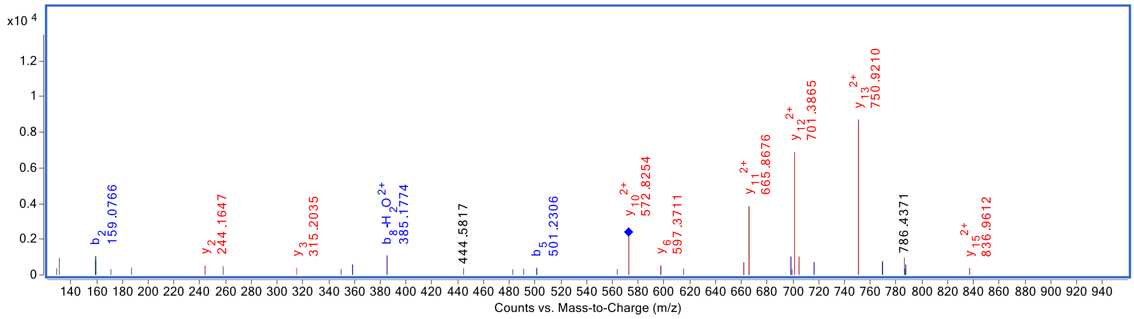

Figure S7 The MS/MS spectra of corresponding peptide without LC-N30 deamidation in P1.

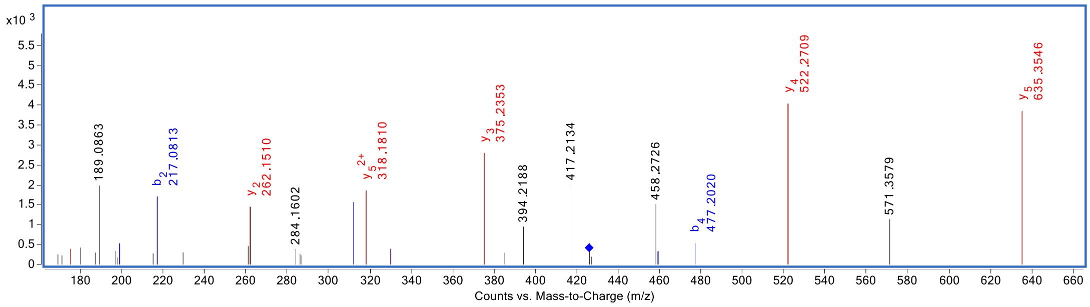

Figure S8 The MS/MS spectra of corresponding peptide with HC-M255 oxidation in P4.

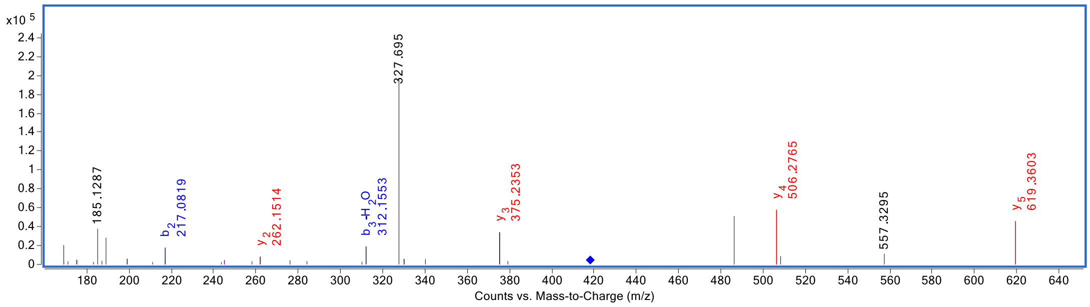

Figure S9 The MS/MS spectra of corresponding peptide without HC-M255 oxidation in P4.

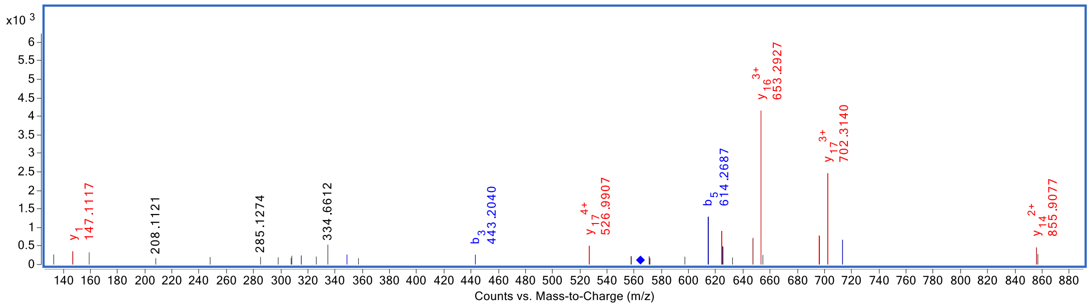

Figure S10 The MS/MS spectra of corresponding peptide with HC-M431 oxidation in P4.

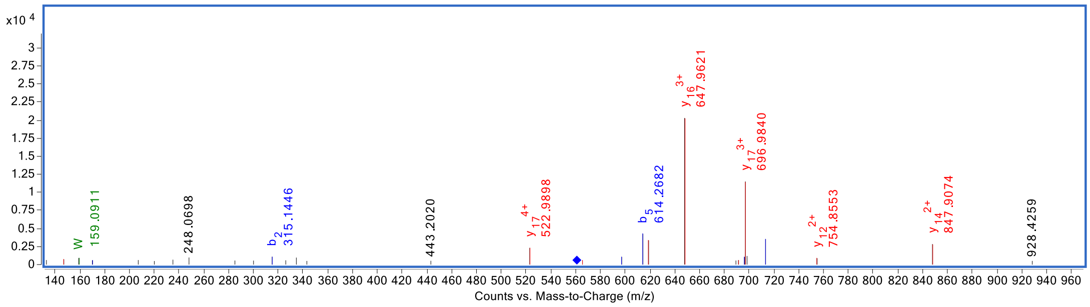

Figure S11 The MS/MS spectra of corresponding peptide without HC-M431 oxidation in P4.

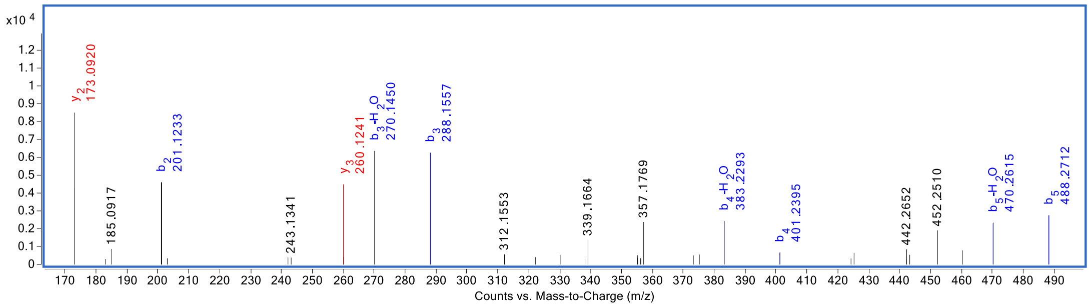

Figure S12 The MS/MS spectra of corresponding peptide with HC C-terminal K loss in P5.

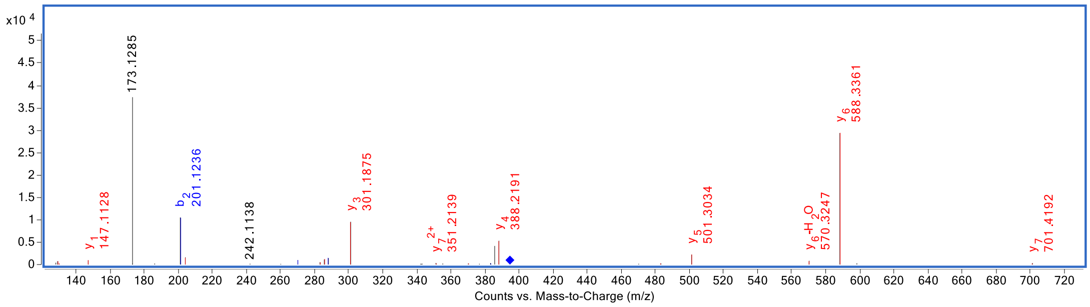

Figure S13 The MS/MS spectra of corresponding peptide without HC C-terminal K loss in P5.

Table S1. The reproducibility of RT in CEX in triplicate.

RT / min	Repeat-1	Repeat-2	Repeat-3	RSD%
P1	8.992	9.040	9.061	0.39
P2	13.009	13.053	13.074	0.25
P3	15.581	15.625	15.640	0.20
P4	17.263	17.308	17.319	0.17
P5	20.043	20.088	20.093	0.14
P6	24.175	24.217	24.216	0.10

Table S2. The reproducibility of mass accuracy of main peak in MS in triplicate.

MS / Da	Repeat-1	Repeat-2	Repeat-3	RSD%
Main Peak	148058.1432	148059.3180	148057.4636	0.0006

Table S3. The sequence coverage of each peak collection in off-line 2D-LC-MS.

Peak collection	P1	P2	P3	P4	P5	P6
**Coverage**	100.00%	99.40%	98.95%	99.55%	99.40%	99.10%

Table S4. The details of identified peptides digested from P1 in off-line 2D-LC-MS.

RT	Seq Loc	Sequence	Pred Mods	Mods	Tgt Seq Mass	Mass	Diff (Bio, ppm)	m/z	Vol	MS/MS Count
**28.996**	A(1-18)	DIQMTQSPSSLSASVGDR	/	/	1877.8789	1877.8792	0.18	626.9673	3303849	2
**33.917**	A(1-24)	DIQMTQSPSSLSASVGDRVTITCR	/	Alkylation (iodoacetamide)(A23)	2608.2585	2608.2562	-0.88	870.4257	105417	
**12.804**	A(19-24)	VTITCR	/	Alkylation (iodoacetamide)(A23)	748.3902	748.3910	1.12	375.2028	2774577	2
**23.190**	A(25-45)	ASQDVNTAVAWYQQKPGKAPK	1*Deamidation(+0.984016)A30	/	2287.1597	2287.1618	0.90	763.4000	206115	1
**24.208**	A(25-45)	ASQDVNTAVAWYQQKPGKAPK	/	/	2286.1757	2286.1803	2.04	572.5500	531247	1
**25.168**	A(25-45)	ASQDVNTAVAWYQQKPGKAPK	1*Deamidation(+0.984016)A30	/	2287.1597	2287.1643	2.01	1144.5900	3359572	1
**24.269**	A(25-45)	ASQDVNTAVAWYQQKPGKAPK	/	/	2286.1757	2286.1802	1.97	1144.1000	4335811	1
**24.252**	A(25-45)	ASQDVNTAVAWYQQKPGKAPK	/	/	2286.1757	2286.1792	1.56	572.5500	1794401	1
**45.384**	A(46-61)	LLIYSASFLYSGVPSR	/	/	1771.9509	1771.9548	2.19	886.9840	62991932	3
**44.373**	A(46-66)	LLIYSASFLYSGVPSRFSGSR	/	/	2306.2059	2306.2121	2.69	769.7447	59683496	3
**6.871**	A(62-66)	FSGSR	/	/	552.2656	552.2706	9.06	277.1426	298775	2
**47.814**	A(67-103)	SGTDFTLTISSLQPEDFATYYCQQHYTT PPTFGQGTK	/	Alkylation (iodoacetamide)(A88)	4186.9106	4186.9267	3.84	1047.7379	247347	2
**46.865**	A(67-107)	SGTDFTLTISSLQPEDFATYYCQQHYTT PPTFGQGTKVEIK	/	Alkylation (iodoacetamide)(A88)	4656.2007	4656.2097	1.94	1165.0588	3166166	4
**6.276**	A(104-107)	VEIK	/	/	487.3006	487.3008	0.44	488.3083	70275	1
**4.473**	A(104-108)	VEIKR	/	/	643.4017	643.4024	1.05	322.7086	1507602	2
**39.739**	A(108-126)	RTVAAPSVFIFPPSDEQLK	/	/	2101.1208	2101.1336	6.11	701.3850	1863176	3
**55.225**	A(108-142)	RTVAAPSVFIFPPSDEQLKSGTASVVCL LNNFYPR	/	Alkylation (iodoacetamide)(A134)	3879.9982	3880.0063	2.08	971.0073	1934551	2
**44.031**	A(109-126)	TVAAPSVFIFPPSDEQLK	/	/	1945.0197	1945.0241	2.26	649.3488	27904074	2

**58.358**	A(109-142)	TVAAPSVFIFPPSDEQLKSGTASVVCLLNNFYPR	/	Alkylation (iodoacetamide)(A134)	3723.8971	3723.9039	1.83	931.9829	24452874	7
**55.596**	A(109-145)	TVAAPSVFIFPPSDEQLKSGTASVVCLLNNFYPREAK	/	Alkylation (iodoacetamide)(A134)	4052.0718	4052.0763	1.11	1014.0259	5611978	4
**47.287**	A(127-142)	SGTASVVCLLNNFYPR	/	Alkylation (iodoacetamide)(A134)	1796.8880	1796.8867	-0.73	899.4502	274005	1
**43.569**	A(127-145)	SGTASVVCLLNNFYPREAK	/	Alkylation (iodoacetamide)(A134)	2125.0626	2125.0670	2.07	709.3630	1639978	2
**43.688**	A(127-145)	SGTASVVCLLNNFYPREAK	/	Alkylation (iodoacetamide)(A134)	2125.0626	2125.0664	1.76	709.3628	3154065	3
**12.577**	A(143-149)	EAKVQWK	/	/	887.4865	887.4863	-0.28	444.7506	315126	1
**25.617**	A(143-169)	EAKVQWKVDNALQSGNSQESVTEQDSK	/	/	3004.4374	3004.4430	1.88	752.1179	181805	1
**11.208**	A(146-149)	VQWK	/	/	559.3118	559.3132	2.40	560.3204	801353	2
**26.756**	A(146-169)	VQWKVDNALQSGNSQESVTEQDSK	/	/	2676.2627	2676.2573	-2.02	893.0914	1397493	2
**36.393**	A(146-183)	VQWKVDNALQSGNSQESVTEQDSKDSTYSLSSTLTLSK	/	/	4160.0033	4160.0131	2.34	1041.0103	13636570	4
**18.086**	A(150-169)	VDNALQSGNSQESVTEQDSK	/	/	2134.9615	2134.9636	0.98	712.6619	379301	2
**36.396**	A(150-188)	VDNALQSGNSQESVTEQDSKDSTYSLSSTLTLSKADYEK	/	/	4224.9670	4224.9431	-5.66	1057.4941	169806	
**28.769**	A(170-190)	DSTYSLSSTLTLSKADYEKHK	/	/	2373.1700	2373.1764	2.71	594.3008	155197	1
**21.032**	A(184-207)	ADYEKHKVYACEVTHQGLSSPVTK	/	Alkylation (iodoacetamide)(A194)	2746.3385	2746.3494	3.98	550.2775	28128150	7
**19.596**	A(189-207)	HKVYACEVTHQGLSSPVTK	/	Alkylation (iodoacetamide)(A194)	2140.0735	2140.0759	1.09	536.0265	5186073	5
**23.790**	A(191-207)	VYACEVTHQGLSSPVTK	/	Alkylation (iodoacetamide)(A194)	1874.9197	1874.9265	3.65	625.9827	7973119	4
**26.619**	A(191-211)	VYACEVTHQGLSSPVTKSFNR	/	Alkylation (iodoacetamide)(A194)	2379.1641	2379.1592	-2.08	595.7964	31109	
**27.027**	A(191-214)	VYACEVTHQGLSSPVTKSFNRGEC	/	Alkylation (iodoacetamide)(A194); Alkylation (iodoacetamide)(A214)	2725.2588	2725.2564	-0.91	682.3220	229135	3
**2.889**	A(208-211)	SFNR	/	/	522.2550	522.2553	0.50	523.2626	159928	1

**6.000**	A(208-214)	SFNRGEC	/	Alkylation (iodoacetamide)(A214)	868.3498	868.3513	1.82	435.1831	6720784	3
**33.913**	B(1-19)	EVQLVESGGGLVQPGGSLR	/	/	1880.9956	1880.9989	1.75	628.0069	51276084	3
**41.416**	B(1-30)	EVQLVESGGGLVQPGGSLRLSCAASGF NIK	/	Alkylation (iodoacetamide)(B22)	3029.5604	3029.5613	0.29	758.3976	202027	3
**44.354**	B(1-38)	EVQLVESGGGLVQPGGSLRLSCAASGF NIKDTYIHWVR	/	Alkylation (iodoacetamide)(B22)	4100.0902	4100.1000	2.40	821.0273	8360391	3
**25.473**	B(20-30)	LSCAASGFNIK	/	Alkylation (iodoacetamide)(B22)	1166.5754	1166.5777	1.97	584.2961	1419811	1
**36.885**	B(20-38)	LSCAASGFNIKDTYIHWVR	/	Alkylation (iodoacetamide)(B22)	2237.1052	2237.1085	1.48	560.2850	102922360	3
**28.010**	B(31-38)	DTYIHWVR	/	/	1088.5403	1088.5420	1.58	545.2779	863859	2
**20.137**	B(39-43)	QAPGK	/	/	499.2754	499.2756	0.25	500.2828	323382	1
**24.544**	B(39-50)	QAPGKGLEWVAR	/	/	1310.7095	1310.7122	2.07	437.9112	53970636	3
**35.400**	B(39-59)	QAPGKGLEWVARIYPTNGYTR	/	/	2376.2339	2376.2363	1.02	595.0677	91717	1
**26.998**	B(44-50)	GLEWVAR	/	/	829.4446	829.4464	2.09	415.7306	3129328	2
**18.048**	B(51-59)	IYPTNGYTR	/	/	1083.5349	1083.5364	1.34	542.7755	18250316	2
**25.021**	B(51-65)	IYPTNGYTRYADSVK	/	/	1746.8577	1746.8598	1.22	583.2936	206940	2
**23.126**	B(51-67)	IYPTNGYTRYADSVKGR	/	/	1959.9803	1959.9846	2.20	491.0035	60065	
**24.312**	B(60-65)	YADSVK	/	/	681.3334	681.3314	-2.93	682.3395	12797600	3
**3.658**	B(60-67)	YADSVKGR	/	/	894.4559	894.4582	2.52	448.2365	1894656	4
**22.377**	B(60-76)	YADSVKGRFTISADTSK	/	/	1844.9268	1844.9308	2.14	462.2402	1289022	3
**18.041**	B(66-76)	GRFTISADTSK	/	/	1181.6041	1181.6056	1.29	591.8100	837930	2
**20.527**	B(68-76)	FTISADTSK	/	/	968.4815	968.4843	2.87	485.2494	3207221	3
**34.635**	B(68-87)	FTISADTSKNTAYLQMNSLR	/	/	2260.1158	2260.1197	1.74	566.0379	2289159	2
**37.168**	B(68-98)	FTISADTSKNTAYLQMNSLRAEDTAVY YCSR	/	Alkylation (iodoacetamide)(B96)	3575.6661	3575.6727	1.86	894.9258	11667746	4
**28.607**	B(77-87)	NTAYLQMNSLR	/	/	1309.6449	1309.6464	1.15	655.8306	2022393	2

**33.713**	B(77-98)	NTAYLQMNSLRAEDTAVYYCSR	/	Alkylation (iodoacetamide)(B96)	2625.1952	2625.1761	-7.26	876.0648	1570025	3
**18.605**	B(88-98)	AEDTAVYYCSR	/	Alkylation (iodoacetamide)(B96)	1333.5609	1333.5636	2.07	667.7892	4421618	3
**48.349**	B(99-124)	WGGDGFYAMDYWGQGTLVTVSSAST K	/	/	2783.2537	2783.2570	1.18	928.7597	2480774	4
**52.162**	B(99-136)	WGGDGFYAMDYWGQGTLVTVSSAST KGPSVFPLAPSSK	/	/	3950.8826	3950.8890	1.62	1317.9693	14832729	5
**51.807**	B(99-150)	WGGDGFYAMDYWGQGTLVTVSSAST KGPSVFPLAPSSKSTSGGTAALGCLVK	/	Alkylation (iodoacetamide)(B147)	5253.5427	5253.5487	1.13	1051.7165	347523	1
**39.404**	B(125-150)	GPSVFPLAPSSKSTSGGTAALGCLVK	/	Alkylation (iodoacetamide)(B147)	2488.2996	2488.3062	2.65	830.4408	429008	2
**28.112**	B(137-150)	STSGGTAALGCLVK	/	Alkylation (iodoacetamide)(B147)	1320.6708	1320.6734	1.97	661.3441	15704163	3
**55.915**	B(137-213)	STSGGTAALGCLVKDYFPEPVTVSWNS GALTSGVHTFPAVLQSSGLYSLSSVVT VPSSSLGTQTYICNVNHKPSNTK	/	Alkylation (iodoacetamide)(B147); Alkylation (iodoacetamide)(B203)	8014.9674	8014.9860	2.32	1336.8470	573866	1
**55.142**	B(137-216)	STSGGTAALGCLVKDYFPEPVTVSWNS GALTSGVHTFPAVLQSSGLYSLSSVVT VPSSSLGTQTYICNVNHKPSNTKVDK	/	Alkylation (iodoacetamide)(B147); Alkylation (iodoacetamide)(B203)	8357.1577	8357.1778	2.40	836.8255	1995931	10
**54.275**	B(151-213)	DYFPEPVTVSWNSGALTSGVHTFPAVL QSSGLYSLSSVVTVPSSSLGTQTYICNV NHKPSNTK	/	Alkylation (iodoacetamide)(B203)	6712.3072	6712.3107	0.52	1343.4705	230882	
**53.230**	B(151-216)	DYFPEPVTVSWNSGALTSGVHTFPAVL QSSGLYSLSSVVTVPSSSLGTQTYICNV NHKPSNTKVDK	/	Alkylation (iodoacetamide)(B203)	7054.4975	7054.5075	1.41	1176.7538	431742	4
**52.292**	B(151-217)	DYFPEPVTVSWNSGALTSGVHTFPAVL QSSGLYSLSSVVTVPSSSLGTQTYICNV NHKPSNTKVDKK	/	Alkylation (iodoacetamide)(B203)	7182.5925	7182.6012	1.21	1198.1091	630332	1
**8.455**	B(214-216)	VDK	/	/	360.2009	360.2013	1.18	361.2086	700526	1

**33.543**	B(218-221)	VEPK	/	/	471.2693	471.2701	1.63	472.2774	35809	
**41.693**	B(222-251)	SCDKTHTCPPCPAPELLGGPSVFLFPPK PK	/	Alkylation (iodoacetamide)(B223); Alkylation (iodoacetamide)(B229); Alkylation (iodoacetamide)(B232)	3333.6349	3333.6398	1.48	667.7353	21017282	5
**43.440**	B(226-251)	THTCPPCPAPELLGGPSVFLFPPKPK	/	Alkylation (iodoacetamide)(B229); Alkylation (iodoacetamide)(B232)	2843.4503	2843.4580	2.70	711.8685	847308	1
**42.134**	B(222-258)	SCDKTHTCPPCPAPELLGGPSVFLFPPK PKDTLMISR	1*Oxidation (M)(+15.994915)B255	Alkylation (iodoacetamide)(B223); Alkylation (iodoacetamide)(B229); Alkylation (iodoacetamide)(B232)	4166.0461	4166.0500	0.92	834.2200	285954	1
**43.405**	B(222-258)	SCDKTHTCPPCPAPELLGGPSVFLFPPK PKDTLMISR	/	Alkylation (iodoacetamide)(B223); Alkylation (iodoacetamide)(B229); Alkylation (iodoacetamide)(B232)	4150.0512	4150.0534	0.51	831.0200	11737881	1
**45.301**	B(226-258)	THTCPPCPAPELLGGPSVFLFPPKPKDT LMISR	/	Alkylation (iodoacetamide)(B229); Alkylation (iodoacetamide)(B232)	3659.8666	3659.8830	4.46	732.9800	15157	
**45.044**	B(226-258)	THTCPPCPAPELLGGPSVFLFPPKPKDT LMISR	/	Alkylation (iodoacetamide)(B229); Alkylation (iodoacetamide)(B232)	3659.8666	3659.8727	1.64	732.9800	69378	1
**53.271**	B(226-277)	THTCPPCPAPELLGGPSVFLFPPKPKDT LMISRTPEVTCVVVDVSHEDPEVK	/	Alkylation (iodoacetamide)(B229); Alkylation (iodoacetamide)(B232); Alkylation (iodoacetamide)(B264)	5594.7598	5594.7940	6.12	933.6400	29946	
**18.488**	B(252-258)	DTLMISR	1*Oxidation (M)(+15.994915)B255	/	850.4219	850.4225	0.75	426.2200	175979	1
**22.042**	B(252-258)	DTLMISR	/	/	834.4269	834.4264	-0.67	835.4300	4775973	1
**39.748**	B(252-277)	DTLMISRTPEVTCVVVDVSHEDPEVK	/	Alkylation (iodoacetamide)(B264)	2954.4365	2954.4547	6.16	739.6200	587178	1
**33.538**	B(259-277)	TPEVTCVVVDVSHEDPEVK	/	Alkylation (iodoacetamide)(B264)	2138.0202	2138.0242	1.90	1070.0192	5994464	2
**42.318**	B(259-291)	TPEVTCVVVDVSHEDPEVKFNWYVDG VEVHNAK	/	Alkylation (iodoacetamide)(B264)	3796.8043	3796.8186	3.77	760.3713	7693752	6
**27.452**	B(278-295)	FNWYVDGVEVHNAKTKPR	/	/	2159.0912	2159.0942	1.37	540.7806	230037	3

**10.523**	B(292-304)	TKPREEQYNSTYR	/	G0F(B300)	3115.3351	3115.3388	1.17	779.8410	227324	2
**43.927**	B(296-323)	EEQYNSTYRVVSVLTVLHQDWLNGKE YK	/	G0F(B300)	4842.2282	4842.2389	2.23	969.8558	658805	
**45.972**	B(305-320)	VVSVLTVLHQDWLNGK	/	/	1806.9992	1807.0023	1.73	603.3413	3084596	3
**43.040**	B(305-323)	VVSVLTVLHQDWLNGKEYK	/	/	2227.2001	2227.2049	2.16	557.8085	58840084	5
**28.107**	B(321-329)	EYKCKVSNK	/	Alkylation (iodoacetamide)(B324)	1154.5754	1154.5694	-5.19	578.2962	87246	
**18.743**	B(324-337)	CKVSNKALPAPIEK	/	Alkylation (iodoacetamide)(B324)	1553.8600	1553.8633	2.14	518.9616	5503470	3
**35.416**	B(326-329)	VSNK	/	/	446.2489	446.2499	2.32	447.2572	172090	1
**20.136**	B(326-337)	VSNKALPAPIEK	/	/	1265.7343	1265.7364	1.66	422.9195	9004415	2
**24.055**	B(326-341)	VSNKALPAPIEKTISK	/	/	1694.9931	1694.9997	3.90	566.0069	835351	4
**22.028**	B(330-337)	ALPAPIEK	/	/	837.4960	837.4981	2.53	419.7564	34757972	2
**26.424**	B(330-341)	ALPAPIEKTISK	/	/	1266.7547	1266.7577	2.37	423.2601	2124079	2
**25.668**	B(330-343)	ALPAPIEKTISKAK	/	/	1465.8868	1465.8899	2.10	489.6371	907550	3
**36.403**	B(338-341)	TISK	/	/	447.2693	447.2696	0.74	448.2772	10854	
**25.683**	B(338-343)	TISKAK	/	/	646.4014	646.4015	0.23	647.4091	16892	
**24.264**	B(344-358)	GQPREPQVYTLPPSR	/	/	1723.9006	1723.9033	1.57	862.9595	55327	1
**24.298**	B(344-363)	GQPREPQVYTLPPSREEMTK	/	/	2342.1689	2342.1698	0.38	781.7298	646273	1
**36.048**	B(348-373)	EPQVYTLPPSREEMTKNQVSLTCLVK	/	Alkylation (iodoacetamide)(B370)	3046.5468	3046.5517	1.62	762.6449	3295984	1
**30.178**	B(359-373)	EEMTKNQVSLTCLVK	/	Alkylation (iodoacetamide)(B370)	1778.8907	1778.8935	1.57	593.9720	2397478	2
**30.934**	B(364-373)	NQVSLTCLVK	/	Alkylation (iodoacetamide)(B370)	1160.6223	1160.6248	2.14	581.3198	24408080	2
**43.459**	B(359-395)	EEMTKNQVSLTCLVKGFYPSDIAVEWE SNGQPENNYK	1*Deamidation(+0.984016)B387/B392/B 393	Alkylation (iodoacetamide)(B370)	4286.9777	4286.9724	-1.24	858.4000	275963	
**43.485**	B(359-395)	EEMTKNQVSLTCLVKGFYPSDIAVEWE SNGQPENNYK	2*Deamidation(+0.984016)B387B392B3 93	/	4230.9402	4230.9677	6.49	1412.3400	28318	

**43.459**	B(359-395)	EEMTKNQVSLTCLVKGFYPSDIAVEWE SNGQPENNYK	3*Deamidation(+0.984016)B387B392B3 93	Alkylation (iodoacetamide)(B370)	4306.9563	4306.9421	-3.27	862.6000	76234	
**43.320**	B(359-395)	EEMTKNQVSLTCLVKGFYPSDIAVEWE SNGQPENNYK	3*Deamidation(+0.984016)B387B392B3 93	/	4231.9242	4231.9569	7.72	1059.2500	16385	
**42.704**	B(374-395)	GFYPSDIAVEWESNGQPENNYK	3*Deamidation(+0.984016)B387B392B3 93	/	2546.0762	2546.0670	-3.59	849.6900	27400	
**40.149**	B(374-395)	GFYPSDIAVEWESNGQPENNYK	1*Deamidation(+0.984016)B387/B392/B 393	/	2416.0132	2416.0336	8.47	605.0200	48325	
**43.405**	B(374-412)	GFYPSDIAVEWESNGQPENNYKTTPPV LDSDGSFFLYSK	/	/	4269.9331	4269.9694	8.48	1068.5000	48336	
**36.416**	B(374-412)	GFYPSDIAVEWESNGQPENNYKTTPPV LDSDGSFFLYSK	3*Deamidation(+0.984016)B387B392B3 93	/	4272.8852	4272.8815	-0.87	1069.4700	52147	
**49.484**	B(374-412)	GFYPSDIAVEWESNGQPENNYKTTPPV LDSDGSFFLYSK	1*Deamidation(+0.984016)B387/B392/B 393	/	4399.0121	4399.0358	5.38	1100.7700	2464443	1
**30.936**	B(364-373)	NQVSLTCLVK	/	Alkylation (iodoacetamide)(B370)	1160.6223	1160.6248	2.13	581.3200	12588779	1
**42.064**	B(396-412)	TTPPVLDSDGSFFLYSK	/	/	1872.9146	1872.9170	1.29	937.4654	7639271	2
**44.306**	B(396-417)	TTPPVLDSDGSFFLYSKLTVDK	/	/	2429.2366	2429.2366	-0.02	810.7528	1155630	1
**41.501**	B(396-419)	TTPPVLDSDGSFFLYSKLTVDKSR	/	/	2672.3698	2672.3738	1.53	669.1010	3291319	2
**8.454**	B(413-417)	LTVDK	/	/	574.3326	574.3332	1.06	575.3402	1353547	2
**4.987**	B(413-419)	LTVDKSR	/	/	817.4658	817.4663	0.61	409.7404	4937024	2
**4.988**	B(418-419)	SR	/	/	261.1437	261.1436	-0.53	262.1508	230120	1
**31.779**	B(413-442)	LTVDKSRWQQGNVFSCSVMHEALHNH YTQK	/	Alkylation (iodoacetamide) (B428)	3599.7150	3599.7396	6.82	720.9600	148339	1
**29.800**	B(418-442)	SRWQQGNVFSCSVMHEALHNHYTQK	/	Alkylation (iodoacetamide) (B428)	3043.3930	3043.3930	0.00	761.8500	1002168	1
**31.616**	B(420-442)	WQQGNVFSCSVMHEALHNHYTQK	/	Alkylation (iodoacetamide) (B428)	2800.2598	2800.2570	-1.03	701.0700	242112	1
**31.331**	B(420-442)	WQQGNVFSCSVMHEALHNHYTQK	/	Alkylation (iodoacetamide) (B428)	2800.2598	2800.2643	1.59	701.0700	15650059	1

**26.406**	B(420-442)	WQQGNVFSCSVMHEALHNHYTQK	1*Oxidation (M)(+15.994915)B431	Alkylation (iodoacetamide) (B428)	2816.2548	2816.2676	4.55	705.0700	253155	1
**36.565**	B(420-450)	WQQGNVFSCSVMHEALHNHYTQKSLS LSPGK	/	Alkylation (iodoacetamide) (B428)	3441.5983	3441.6043	1.75	689.3300	74111	1
**24.305**	B(443-449)	SLSLSPG	Lys-loss K450	/	659.3490	659.3499	1.31	660.3600	17084651	1
**19.281**	B(443-450)	SLSLSPGK	/	/	787.4440	787.4445	0.70	394.7300	452357	1

A: LC B: HC

Table S5. The details of identified peptides digested from P2 in off-line 2D-LC-MS.

RT	Seq Loc	Sequence	Pred Mods	Mods	Tgt Seq Mass	Mass	Diff (Bio, ppm)	m/z	Vol	MS/MS Count
**28.923**	A(1-18)	DIQMTQSPSSLSASVGDR	/	/	1877.8789	1877.8822	1.75	939.9481	41657884	5
**33.453**	A(1-24)	DIQMTQSPSSLSASVGDRVTITCR	/	Alkylation (iodoacetamide)(A23)	2608.2585	2608.2637	1.98	870.4285	7563221	3
**12.673**	A(19-24)	VTITCR	/	Alkylation (iodoacetamide)(A23)	748.3902	748.3912	1.36	375.2031	39450856	3
**44.474**	A(25-61)	ASQDVNTAVAWYQQKPGKAPKLLI YSASFLYSGVPSR	/	/	4040.1160	4040.1367	5.14	809.0400	272544	1
**23.174**	A(25-45)	ASQDVNTAVAWYQQKPGKAPK	1*Deamidation(+0.984016)A30	/	2287.1597	2287.1667	3.05	572.8000	486617	1
**24.320**	A(25-45)	ASQDVNTAVAWYQQKPGKAPK	/	/	2286.1757	2286.1811	2.37	572.5500	462574	1
**24.168**	A(25-45)	ASQDVNTAVAWYQQKPGKAPK	/	/	2286.1757	2286.1802	1.97	763.0700	16941114	1
**24.791**	A(25-42)	ASQDVNTAVAWYQQKPGK	1*Deamidation(+0.984016)A30	/	1990.9749	1990.9810	3.08	664.6700	43069	1
**26.886**	A(25-42)	ASQDVNTAVAWYQQKPGK	1*Deamidation(+0.984016)A30	/	1990.9749	1990.9765	0.82	664.6700	97651	1
**25.933**	A(25-42)	ASQDVNTAVAWYQQKPGK	/	/	1989.9908	1989.9963	2.74	996.0000	1122393	1
**43.665**	A(19-45)	VTITCRASQDVNTAVAWYQQKPGK APK	1*Deamidation(+0.984016)A30	Alkylation (iodoacetamide)(A23)	2889.4443	2889.4252	-6.63	964.1500	21256	/
**28.003**	A(19-42)	VTITCRASQDVNTAVAWYQQKPGK	1*Deamidation(+0.984016)A30	Alkylation (iodoacetamide)(A23)	2664.3330	2664.3156	-6.53	889.1100	23310	/
**43.626**	A(43-61)	APKLLIYSASFLYSGVPSR	/	/	2068.1357	2068.1473	5.62	690.3910	33230	/
**45.341**	A(46-61)	LLIYSASFLYSGVPSR	/	/	1771.9509	1771.9586	4.37	886.9854	150476368	3
**44.423**	A(46-66)	LLIYSASFLYSGVPSRFSGSR	/	/	2306.2059	2306.2148	3.86	769.7455	4539252	4
**45.695**	A(62-103)	FSGSRSGTDFTLTISSLQPEDFATYYC QQHYTTPPTFGQGTK	/	Alkylation (iodoacetamide)(A88)	4721.1657	4721.1764	2.28	1181.3003	236142	1
**46.831**	A(67-107)	SGTDFTLTISSLQPEDFATYYCQQHY TTPPTFGQGTKVEIK	/	Alkylation (iodoacetamide)(A88)	4656.2007	4656.2099	1.98	1165.0584	2340995	6

**6.202**	A(104-107)	VEIK	/	/	487.3006	487.3016	1.96	488.3088	1524726	2
**4.254**	A(104-108)	VEIKR	/	/	643.4017	643.4034	2.66	322.7090	15733585	3
**40.371**	A(108-126)	RTVAAPSVFIFPPSDEQLK	/	/	2101.1208	2101.1284	3.64	701.3830	1153858	1
**43.986**	A(109-126)	TVAAPSVFIFPPSDEQLK	/	/	1945.0197	1945.0249	2.70	649.3489	119913176	3
**58.413**	A(109-142)	TVAAPSVFIFPPSDEQLKSGTASVVC LLNNFYPR	/	Alkylation (iodoacetamide)(A134)	3723.8971	3723.9030	1.59	931.9828	2673987	7
**47.095**	A(127-142)	SGTASVVCLLNNFYPR	/	Alkylation (iodoacetamide)(A134)	1796.8880	1796.8930	2.82	899.4538	26450	1
**12.486**	A(143-149)	EAKVQWK	/		887.4865	887.4872	0.77	444.7510	17256	1
**10.976**	A(146-149)	VQWK	/	/	559.3118	559.3129	1.93	560.3201	15206710	2
**26.730**	A(146-169)	VQWKVDNALQSGNSQESVTEQDSK	/	/	2676.2627	2676.2626	-0.03	893.0943	1131121	2
**36.396**	A(146-183)	VQWKVDNALQSGNSQESVTEQDSK DSTYSLSSTLTLSK	/	/	4160.0033	4160.0148	2.76	1041.0107	3599671	4
**18.036**	A(150-169)	VDNALQSGNSQESVTEQDSK	/	/	2134.9615	2134.9650	1.64	712.6623	11271291	4
**33.375**	A(150-183)	VDNALQSGNSQESVTEQDSKDSTYS LSSTLTLSK	/	/	3618.7021	3618.7087	1.84	1207.2432	10369891	2
**33.374**	A(170-183)	DSTYSLSSTLTLSK	/	/	1501.7512	1501.7542	2.00	751.8845	16998546	3
**9.948**	A(184-190)	ADYEKHK	/	/	889.4294	889.4287	-0.72	445.7218	15351	1
**21.050**	A(184-207)	ADYEKHKVYACEVTHQGLSSPVTK	/	Alkylation (iodoacetamide)(A194)	2746.3385	2746.3483	3.57	550.2773	7676058	5
**6.763**	A(189-190)	HK	/	/	283.1644	283.1647	1.05	284.1719	25065	/
**19.385**	A(189-207)	HKVYACEVTHQGLSSPVTK	/	Alkylation (iodoacetamide)(A194)	2140.0735	2140.0794	2.75	536.0272	68009912	7
**23.699**	A(191-207)	VYACEVTHQGLSSPVTK	/	Alkylation (iodoacetamide)(A194)	1874.9197	1874.9238	2.20	625.9819	116770352	4
**2.825**	A(208-211)	SFNR	/	/	522.2550	522.2560	1.78	523.2633	2590695	1
**5.783**	A(208-214)	SFNRGEC	/	Alkylation (iodoacetamide)(A214)	868.3498	868.3513	1.78	435.1829	15893492	3
**33.824**	B(1-19)	EVQLVESGGGLVQPGGSLR	/	/	1880.9956	1880.9986	1.59	941.5050	115337288	3

**44.385**	B(1-38)	EVQLVESGGGLVQPGGSLRLSCAAS GFNIKDTYIHWVR	/	Alkylation (iodoacetamide)(B22)	4100.0902	4100.1001	2.41	821.2285	558772	/
**25.399**	B(20-30)	LSCAASGFNIK	/	Alkylation (iodoacetamide)(B22)	1166.5754	1166.5785	2.62	584.2966	24687512	3
**36.837**	B(20-38)	LSCAASGFNIKDTYIHWVR	/	Alkylation (iodoacetamide)(B22)	2237.1052	2237.1117	2.92	560.2856	165546368	3
**27.868**	B(31-38)	DTYIHWVR	/	/	1088.5403	1088.5435	2.92	545.2792	15680454	3
**24.527**	B(39-50)	QAPGKGLEWVAR	/	/	1310.7095	1310.7131	2.71	437.9118	48018252	3
**26.876**	B(44-50)	GLEWVAR	/	/	829.4446	829.4465	2.19	415.7306	56470056	2
**17.994**	B(51-59)	IYPTNGYTR	/	/	1083.5349	1083.5367	1.69	542.7756	53095848	2
**24.277**	B(60-65)	YADSVK	/	/	681.3334	681.3318	-2.27	682.3396	25100716	3
**3.532**	B(60-67)	YADSVKGR	/	/	894.4559	894.4583	2.61	448.2365	2043049	3
**17.961**	B(66-76)	GRFTISADTSK	/	/	1181.6041	1181.6061	1.71	591.8102	11313114	3
**31.872**	B(66-87)	GRFTISADTSKNTAYLQMNSLR	/	/	2473.2384	2473.2416	1.30	619.3182	7565039	2
**20.454**	B(68-76)	FTISADTSK	/	/	968.4815	968.4845	3.14	485.2499	44877968	3
**34.606**	B(68-87)	FTISADTSKNTAYLQMNSLR	/	/	2260.1158	2260.1204	2.06	754.3806	33521476	5
**28.502**	B(77-87)	NTAYLQMNSLR	/	/	1309.6449	1309.6470	1.64	655.8312	43264108	4
**33.692**	B(77-98)	NTAYLQMNSLRAEDTAVYYCSR	/	Alkylation (iodoacetamide)(B96)	2625.1952	2625.2002	1.91	876.0740	576435	2
**18.511**	B(88-98)	AEDTAVYYCSR	/	Alkylation (iodoacetamide)(B96)	1333.5609	1333.5629	1.54	667.7889	24849990	3
**48.322**	B(99-124)	WGGDGFYAMDYWGQGTLVTVSSA STK	/	/	2783.2537	2783.2610	2.61	928.7610	8847941	3
**52.269**	B(99-136)	WGGDGFYAMDYWGQGTLVTVSSA STKGPSVFPLAPSSK	/	/	3950.8826	3950.8914	2.24	988.7304	937588	2
**31.892**	B(125-136)	GPSVFPLAPSSK	/	/	1185.6394	1185.6424	2.52	1186.6504	11967089	2
**27.958**	B(137-150)	STSGGTAALGCLVK	/	Alkylation (iodoacetamide)(B147)	1320.6708	1320.6736	2.14	661.3444	68547152	3

**55.926**	B(137-213)	STSGGTAALGCLVKDYFPEPVTVSW NSGALTSGVHTFPAVLQSSGLYSLSS VVTVPSSSLGTQTYICNVNHKPSNTK	/	Alkylation (iodoacetamide)(B147); Alkylation (iodoacetamide)(B203)	8014.9674	8014.9796	1.53	891.5610	3142870	6
**55.189**	B(137-216)	STSGGTAALGCLVKDYFPEPVTVSW NSGALTSGVHTFPAVLQSSGLYSLSS VVTVPSSSLGTQTYICNVNHKPSNTK VDK	/	Alkylation (iodoacetamide)(B147); Alkylation (iodoacetamide)(B203)	8357.1577	8357.1874	3.56	836.7206	1878335	13
**54.211**	B(151-213)	DYFPEPVTVSWNSGALTSGVHTFPA VLQSSGLYSLSSVVTVPSSSLGTQTYI CNVNHKPSNTK	/	Alkylation (iodoacetamide)(B203)	6712.3072	6712.3260	2.80	1343.4711	25229012	8
**53.134**	B(151-216)	DYFPEPVTVSWNSGALTSGVHTFPA VLQSSGLYSLSSVVTVPSSSLGTQTYI CNVNHKPSNTKVDK	/	Alkylation (iodoacetamide)(B203)	7054.4975	7054.5160	2.61	1176.7591	14840968	14
**52.299**	B(151-217)	DYFPEPVTVSWNSGALTSGVHTFPA VLQSSGLYSLSSVVTVPSSSLGTQTYI CNVNHKPSNTKVDKK	/	Alkylation (iodoacetamide)(B203)	7182.5925	7182.6196	3.77	1198.1079	8391013	11
**8.295**	B(214-216)	VDK	/	/	360.2009	360.2018	2.65	361.2092	5667102	1
**41.536**	B(222-251)	SCDKTHTCPPCPAPELLGGPSVFLFPP KPK	/	Alkylation (iodoacetamide)(B223); Alkylation (iodoacetamide)(B229); Alkylation (iodoacetamide)(B232)	3333.6349	3333.6437	2.66	667.7363	127802384	5
**43.369**	B(226-251)	THTCPPCPAPELLGGPSVFLFPPKPK	/	Alkylation (iodoacetamide)(B229); Alkylation (iodoacetamide)(B232)	2843.4503	2843.4580	2.73	711.8719	31082982	3
**43.686**	B(222-258)	SCDKTHTCPPCPAPELLGGPSVFLFPP KPKDTLMISR	/	Alkylation (iodoacetamide)(B223); Alkylation (iodoacetamide)(B229); Alkylation (iodoacetamide)(B232)	4150.0512	4150.0685	4.16	831.0200	303714	/

**43.509**	B(222-258)	SCDKTHTCPPCPAPELLGGPSVFLFPPKPKDTLMISR	/	Alkylation (iodoacetamide)(B223); Alkylation (iodoacetamide)(B229); Alkylation (iodoacetamide)(B232)	4150.0512	4150.0646	3.21	831.0200	899561	1
**18.465**	B(252-258)	DTLMISR	1*Oxidation (M)(+15.994915)B255	/	850.4219	850.4237	2.14	851.4300	2862747	1
**21.938**	B(252-258)	DTLMISR	/	/	834.4269	834.4294	2.93	835.4400	30925824	1
**39.711**	B(252-277)	DTLMISRTPEVTCVVVDVSHEDPEVK	/	Alkylation (iodoacetamide)(B264)	2954.4365	2954.4369	0.11	985.8200	25298	/
**45.473**	B(252-291)	DTLMISRTPEVTCVVVDVSHEDPEVKFNWYVDGVEVHNAK	/	Alkylation (iodoacetamide)(B264)	4613.2207	4613.2293	1.88	923.6500	40586	/
**33.443**	B(259-277)	TPEVTCVVVDVSHEDPEVK	/	Alkylation (iodoacetamide)(B264)	2138.0202	2138.0267	3.05	713.6840	127277512	3
**42.311**	B(259-291)	TPEVTCVVVDVSHEDPEVKFNWYVDGVEVHNAK	/	Alkylation (iodoacetamide)(B264)	3796.8043	3796.8060	0.44	760.3684	6969400	5
**31.913**	B(278-291)	FNWYVDGVEVHNAK	/	/	1676.7947	1676.8018	4.23	559.9401	147875	/
**23.815**	B(292-295)	TKPR	/	/	500.3071	500.3076	1.12	501.3150	47904	/
**10.364**	B(292-304)	TKPREEQYNSTYR	/	G0F(B300)	3115.3351	3115.3382	1.00	1039.4529	2863771	5
**11.928**	B(296-304)	EEQYNSTYR	/	G0F(B300)	2633.0386	2633.0445	2.24	1317.5299	200367	1
**43.921**	B(296-323)	EEQYNSTYRVVSVLTVLHQDWLNGKEYK	/	G0F(B300)	4842.2282	4842.2441	3.30	969.4585	374091	/
**45.888**	B(305-320)	VVSVLTVLHQDWLNGK	/	/	1806.9992	1807.0047	3.05	603.3424	142278048	6
**42.884**	B(305-323)	VVSVLTVLHQDWLNGKEYK	/	/	2227.2001	2227.2061	2.67	557.8090	256569984	4
**18.740**	B(324-337)	CKVSNKALPAPIEK	/	Alkylation (iodoacetamide)(B324)	1553.8600	1553.8645	2.95	518.9625	39526	/
**35.390**	B(326-329)	VSNK	/	/	446.2489	446.2494	1.07	447.2566	220453	1
**20.135**	B(326-337)	VSNKALPAPIEK	/	/	1265.7343	1265.7370	2.12	422.9195	372245	2
**21.955**	B(330-337)	ALPAPIEK	/	/	837.4960	837.4979	2.29	419.7564	114876360	2
**26.381**	B(330-341)	ALPAPIEKTISK	/	/	1266.7547	1266.7568	1.66	423.2596	68487	/

**52.070**	B(330-343)	ALPAPIEKTISKAK	/	/	1465.8868	1465.9003	9.18	1466.9103	21705	/
**15.627**	B(338-341)	TISK	/	/	447.2693	447.2696	0.60	448.2767	28952	1
**24.165**	B(344-358)	GQPREPQVYTLPPSR	/	/	1723.9006	1723.9038	1.85	862.9593	587304	1
**24.170**	B(344-363)	GQPREPQVYTLPPSREEMTK	/	/	2342.1689	2342.1751	2.64	586.5514	1424885	1
**25.951**	B(348-358)	EPQVYTLPPSR	/	/	1285.6667	1285.6691	1.90	643.8417	746607	2
**25.976**	B(348-363)	EPQVYTLPPSREEMTK	/	/	1903.9350	1903.9396	2.45	635.6540	17651578	2
**36.073**	B(348-373)	EPQVYTLPPSREEMTKNQVSLTCLV K	/	Alkylation (iodoacetamide)(B370)	3046.5468	3046.5526	1.92	762.6457	5006069	4
**30.137**	B(359-373)	EEMTKNQVSLTCLVK	/	Alkylation (iodoacetamide)(B370)	1778.8907	1778.8938	1.75	593.9722	3779942	4
**30.803**	B(364-373)	NQVSLTCLVK	/	Alkylation (iodoacetamide)(B370)	1160.6223	1160.6265	3.54	581.3207	165628960	3
**40.316**	B(364-412)	NQVSLTCLVKGFYPSDIAVEWESNG QPENNYKTTPPVLDSDGSFFLYSK	2*Deamidation(+0.984016)B387B392B393	Alkylation (iodoacetamide)(B370)	5485.5864	5485.6019	2.83	915.4500	51948	/
**40.316**	B(374-395)	GFYPSDIAVEWESNGQPENNYK	1*Deamidation(+0.984016)B387/B392/B393	/	2544.1081	2544.1185	4.08	849.0500	170580	1
**39.586**	B(374-395)	GFYPSDIAVEWESNGQPENNYK	1*Deamidation(+0.984016)B387/B392/B393	/	2544.1081	2544.1234	6.02	849.0500	239252	1
**39.756**	B(374-395)	GFYPSDIAVEWESNGQPENNYK	/	/	2543.1241	2543.1342	3.99	848.7200	6231989	1
**18.025**	B(374-412)	GFYPSDIAVEWESNGQPENNYKTTP PVLDSDGSFFLYSK	/	/	4269.9331	4269.9309	-0.52	1424.3200	28672	/
**41.936**	B(396-412)	TTPPVLDSDGSFFLYSK	/	/	1872.9146	1872.9183	2.00	937.4662	94685944	3
**41.383**	B(396-417)	TTPPVLDSDGSFFLYSKLTVDK	/	/	2429.2366	2429.2396	1.21	810.7543	179133	/
**41.389**	B(396-419)	TTPPVLDSDGSFFLYSKLTVDKSR	/	/	2672.3698	2672.3737	1.46	669.1007	660090	/
**8.290**	B(413-417)	LTVDK	/	/	574.3326	574.3338	2.11	575.3408	13831522	2
**4.849**	B(413-419)	LTVDKSR	/	/	817.4658	817.4674	2.02	409.7410	4151357	2
**21.952**	B(418-419)	SR	/	/	261.1437	261.1443	2.33	262.1516	1209220	1
**29.662**	B(418-442)	SRWQQGNVFSCSVMHEALHNHYTQ K	/	Alkylation (iodoacetamide) (B428)	3043.3930	3043.4004	2.44	609.6900	1046104	1

**55.502**	B(420-450)	WQQGNVFSCSVMHEALHNHYTQKS LSLSPGK	/	Alkylation (iodoacetamide) (B428)	3569.6933	3569.6777	-4.35	1190.9000	202762	/
**26.302**	B(420-442)	WQQGNVFSCSVMHEALHNHYTQK	1*Oxidation (M)(+15.994915)B431	Alkylation (iodoacetamide) (B428)	2816.2548	2816.2609	2.17	705.0700	476644	1
**31.212**	B(420-442)	WQQGNVFSCSVMHEALHNHYTQK	/	Alkylation (iodoacetamide) (B428)	2800.2598	2800.2662	2.26	561.0600	34195861	1
**24.268**	B(443-449)	SLSLSPG	Lys-loss K450	/	659.3490	659.3513	3.51	330.6800	32215835	1
**19.234**	B(443-450)	SLSLSPGK	/	/	787.4440	787.4477	4.78	788.4500	1628754	1

A: LC B: HC

Table S6. The details of identified peptides digested from P3 in off-line 2D-LC-MS.

RT	Seq Loc	Sequence	Pred Mods	Mods	Tgt Seq Mass	Mass	Diff (Bio, ppm)	m/z	Vol	MS/MS Count
**28.927**	A(1-18)	DIQMTQSPSSLSASVGDR	/	/	1877.8789	1877.8831	2.25	939.9486	67355624	4
**33.492**	A(1-24)	DIQMTQSPSSLSASVGDRVTITCR	/	Alkylation (iodoacetamide)(A23)	2608.2585	2608.2647	2.38	870.4292	2798122	3
**12.671**	A(19-24)	VTITCR	/	Alkylation (iodoacetamide)(A23)	748.3902	748.3918	2.20	375.2031	63590084	3
**44.409**	A(25-61)	ASQDVNTAVAWYQQKPGKAPKLLIYSASFLYSGVPSR	/	/	4040.1160	4040.1255	2.34	809.0300	115229	/
**23.184**	A(25-45)	ASQDVNTAVAWYQQKPGKAPK	1*Deamidation(+0.984016)A30	/	2287.1597	2287.1654	2.48	572.8000	199220	1
**25.117**	A(25-45)	ASQDVNTAVAWYQQKPGKAPK	1*Deamidation(+0.984016)A30	/	2287.1597	2287.1668	3.12	1144.5900	745612	1
**24.165**	A(25-45)	ASQDVNTAVAWYQQKPGKAPK	/	/	2286.1757	2286.1816	2.59	763.0700	15693312	1
**26.899**	A(25-42)	ASQDVNTAVAWYQQKPGK	1*Deamidation(+0.984016)A30	/	1990.9749	1990.9818	3.50	996.5000	283665	1
**25.898**	A(25-42)	ASQDVNTAVAWYQQKPGK	/	/	1989.9908	1989.9957	2.43	664.3400	5822336	1
**24.183**	A(43-45)	APK	/	/	314.1954	314.1966	3.76	315.2038	18301	/
**44.623**	A(43-61)	APKLLIYSASFLYSGVPSR	/	/	2068.1357	2068.1374	0.80	690.3868	142239	/
**45.347**	A(46-61)	LLIYSASFLYSGVPSR	/	/	1771.9509	1771.9557	2.70	886.9847	172026992	3
**52.213**	A(46-103)	LLIYSASFLYSGVPSRFSGSRSGTDFTLTISSLQPEDFATYYCQQHYTTPPTFGQGTK	/	Alkylation (iodoacetamide)(A88)	6475.1060	6475.1228	2.60	1296.0340	100072	/
**45.702**	A(62-103)	FSGSRSGTDFTLTISSLQPEDFATYYCQQHYTTPPTFGQGTK	/	Alkylation (iodoacetamide)(A88)	4721.1657	4721.1780	2.62	1181.3017	652122	2
**47.872**	A(67-103)	SGTDFTLTISSLQPEDFATYYCQQHYTTPPTFGQGTK	/	Alkylation (iodoacetamide)(A88)	4186.9106	4186.9207	2.42	1047.7368	396188	4
**46.858**	A(67-107)	SGTDFTLTISSLQPEDFATYYCQQHYTTPPTFGQGTKVEIK	/	Alkylation (iodoacetamide)(A88)	4656.2007	4656.2136	2.78	932.2494	408597	1

**44.855**	A(67-108)	SGTDFTLTISSLQPEDFATYYCQQHYTTPPTFGQG TKVEIKR	/	Alkylation (iodoacetamide)(A88)	4812.3018	4812.3337	6.64	963.4775	878303	1
**6.198**	A(104-107)	VEIK	/	/	487.3006	487.3017	2.35	488.3091	3338581	2
**4.244**	A(104-108)	VEIKR	/	/	643.4017	643.4036	3.00	322.7092	23570492	2
**43.994**	A(109-126)	TVAAPSVFIFPPSDEQLK	/	/	1945.0197	1945.0243	2.38	649.3491	147060592	3
**49.064**	A(127-142)	SGTASVVCLLNNFYPR	/	Alkylation (iodoacetamide)(A134)	1797.8720	1797.8775	3.09	600.3004	814120	2
**10.973**	A(146-149)	VQWK	/	/	559.3118	559.3132	2.52	560.3205	24059184	2
**33.051**	A(146-169)	VQWKVDNALQSGNSQESVTEQDSK	/	/	2676.2627	2676.2748	4.50	670.0850	90320	/
**36.397**	A(146-183)	VQWKVDNALQSGNSQESVTEQDSKDSTYSLSST LTLSK	/	/	4160.0033	4160.0004	-0.70	1041.0062	227115	1
**18.020**	A(150-169)	VDNALQSGNSQESVTEQDSK	/	/	2134.9615	2134.9657	1.96	1068.4896	19791448	2
**33.097**	A(150-188)	VDNALQSGNSQESVTEQDSKDSTYSLSSTLTLSK ADYEK	/	/	4224.9670	4224.9755	2.01	1057.2513	98342	/
**33.786**	A(170-183)	DSTYSLSSTLTLSK	/	/	1501.7512	1501.7533	1.40	1502.7602	50845	/
**33.043**	A(170-188)	DSTYSLSSTLTLSKADYEK	/	/	2108.0161	2108.0218	2.72	703.6813	38208	/
**21.167**	A(184-207)	ADYEKHKVYACEVTHQGLSSPVTK	/	Alkylation (iodoacetamide)(A194)	2746.3385	2746.3428	1.59	550.2764	489160	2
**6.792**	A(189-190)	HK	/	/	283.1644	283.1644	-0.28	284.1716	26005	1
**19.363**	A(189-207)	HKVYACEVTHQGLSSPVTK	/	Alkylation (iodoacetamide)(A194)	2140.0735	2140.0781	2.15	536.0262	67115704	7
**23.665**	A(191-207)	VYACEVTHQGLSSPVTK	/	Alkylation (iodoacetamide)(A194)	1874.9197	1874.9237	2.18	625.9823	153937600	4
**26.594**	A(191-211)	VYACEVTHQGLSSPVTKSFNR	/	Alkylation (iodoacetamide)(A194)	2379.1641	2379.1701	2.52	595.7999	61512	1
**2.804**	A(208-211)	SFNR	/	/	522.2550	522.2558	1.50	523.2630	5732186	2
**5.797**	A(208-214)	SFNRGEC	/	Alkylation (iodoacetamide)(A214)	868.3498	868.3517	2.27	435.1832	14192123	2
**5.801**	A(212-214)	GEC	/	Alkylation (iodoacetamide)(A214)	364.1053	364.1058	1.42	365.1131	36719	/
**33.829**	B(1-19)	EVQLVESGGGLVQPGGSLR	/	/	1880.9956	1881.0005	2.60	941.5069	119509456	5

**44.398**	B(1-38)	EVQLVESGGGLVQPGGSLRLSCAASGFNIKDTYI HWVR	/	Alkylation (iodoacetamide)(B22)	4100.0902	4100.1071	4.12	821.0317	1053404	1
**25.350**	B(20-30)	LSCAASGFNIK	/	Alkylation (iodoacetamide)(B22)	1166.5754	1166.5782	2.36	584.2965	56668168	4
**36.881**	B(20-38)	LSCAASGFNIKDTYIHWVR	/	Alkylation (iodoacetamide)(B22)	2237.1052	2237.1117	2.91	560.2851	117543664	5
**35.023**	B(20-43)	LSCAASGFNIKDTYIHWVRQAPGK	/	Alkylation (iodoacetamide)(B22)	2718.3700	2718.3829	4.74	680.6027	147025	1
**27.841**	B(31-38)	DTYIHWVR	/	/	1088.5403	1088.5438	3.14	545.2790	36390336	3
**24.595**	B(39-50)	QAPGKGLEWVAR	/	/	1310.7095	1310.7146	3.84	437.9123	7216036	2
**35.414**	B(39-59)	QAPGKGLEWVARIYPTNGYTR	/	/	2376.2339	2376.2393	2.30	595.0668	136731	2
**26.849**	B(44-50)	GLEWVAR	/	/	829.4446	829.4476	3.57	415.7312	87174472	2
**17.994**	B(51-59)	IYPTNGYTR	/	/	1083.5349	1083.5374	2.30	542.7761	57457908	2
**46.822**	B(51-65)	IYPTNGYTRYADSVK	/	/	1746.8577	1746.8667	5.14	583.2959	81448	1
**24.272**	B(60-65)	YADSVK	/	/	681.3334	681.3321	-1.89	682.3398	26078030	3
**3.538**	B(60-67)	YADSVKGR	/	/	894.4559	894.4584	2.78	448.2364	212060	1
**17.966**	B(66-76)	GRFTISADTSK	/	/	1181.6041	1181.6072	2.66	591.8106	14603678	3
**31.901**	B(66-87)	GRFTISADTSKNTAYLQMNSLR	/	/	2473.2384	2473.2448	2.58	619.3190	2097311	2
**20.418**	B(68-76)	FTISADTSK	/	/	968.4815	968.4840	2.55	485.2493	72796392	2
**34.628**	B(68-87)	FTISADTSKNTAYLQMNSLR	/	/	2260.1158	2260.1210	2.30	754.3809	9008060	3
**37.174**	B(68-98)	FTISADTSKNTAYLQMNSLRAEDTAVYYCSR	/	Alkylation (iodoacetamide)(B96)	3575.6661	3575.6689	0.78	894.9254	481546	1
**28.493**	B(77-87)	NTAYLQMNSLR	/	/	1309.6449	1309.6481	2.45	655.8315	66727356	4
**33.711**	B(77-98)	NTAYLQMNSLRAEDTAVYYCSR	/	Alkylation (iodoacetamide)(B96)	2625.1952	2625.2014	2.35	876.0749	130811	/
**18.501**	B(88-98)	AEDTAVYYCSR	/	Alkylation (iodoacetamide)(B96)	1333.5609	1333.5629	1.54	667.7891	29302908	3
**48.319**	B(99-124)	WGGDGFYAMDYWGQGTLVTVSSASTK	/	/	2783.2537	2783.2615	2.77	928.7612	10177136	3
**52.246**	B(99-136)	WGGDGFYAMDYWGQGTLVTVSSASTKGPSVFP LAPSSK	/	/	3950.8826	3950.8925	2.51	988.7321	421850	1
**31.923**	B(125-136)	GPSVFPLAPSSK	/	/	1185.6394	1185.6426	2.71	593.8287	148294816	3

**39.556**	B(125-150)	GPSVFPLAPSSKSTSGGTAALGCLVK	/	Alkylation (iodoacetamide)(B147)	2488.2996	2488.3072	3.07	830.4425	146153	1
**27.948**	B(137-150)	STSGGTAALGCLVK	/	Alkylation (iodoacetamide)(B147)	1320.6708	1320.6741	2.55	661.3445	48932852	3
**54.194**	B(151-213)	DYFPEPVTVSWNSGALTSGVHTFPAVLQSSGLYS LSSVVTVPSSSLGTQTYICNVNHKPSNTK	/	Alkylation (iodoacetamide)(B203)	6712.3072	6712.3288	3.23	1343.4722	39645656	16
**53.188**	B(151-216)	DYFPEPVTVSWNSGALTSGVHTFPAVLQSSGLYS LSSVVTVPSSSLGTQTYICNVNHKPSNTKVDK	/	Alkylation (iodoacetamide)(B203)	7054.4975	7054.5117	2.01	1176.7585	8358480	10
**52.226**	B(151-217)	DYFPEPVTVSWNSGALTSGVHTFPAVLQSSGLYS LSSVVTVPSSSLGTQTYICNVNHKPSNTKVDKK	/	Alkylation (iodoacetamide)(B203)	7182.5925	7182.6192	3.72	1198.1114	3927637	7
**41.602**	B(222-251)	SCDKTHTCPPCPAPELLGGPSVFLFPPKPK	/	Alkylation (iodoacetamide)(B223); Alkylation (iodoacetamide)(B229); Alkylation (iodoacetamide)(B232)	3333.6349	3333.6453	3.14	667.7367	102824888	5
**43.358**	B(226-251)	THTCPPCPAPELLGGPSVFLFPPKPK	/	Alkylation (iodoacetamide)(B229); Alkylation (iodoacetamide)(B232)	2843.4503	2843.4599	3.37	711.8720	78297216	4
**45.488**	B(252-291)	DTLMISRTPEVTCVVVDVSHEDPEVKFNWYVDG VEVHNAK	/	Alkylation (iodoacetamide)(B264)	4613.2207	4613.2221	0.30	923.8500	67565	/
**39.749**	B(252-277)	DTLMISRTPEVTCVVVDVSHEDPEVK	/	Alkylation (iodoacetamide)(B264)	2954.4365	2954.4440	2.54	985.8200	21794	/
**18.472**	B(252-258)	DTLMISR	1*Oxidation (M)(+15.994915)B255	/	850.4219	850.4210	-1.02	851.4300	1606432	1
**21.917**	B(252-258)	DTLMISR	/	/	834.4269	834.4299	3.49	835.4400	33810533	1
**45.315**	B(226-258)	THTCPPCPAPELLGGPSVFLFPPKPKDTLMISR	/	Alkylation (iodoacetamide)(B229); Alkylation (iodoacetamide)(B232)	3602.8452	3602.8316	-3.78	1202.2900	63446	/
**43.529**	B(222-258)	SCDKTHTCPPCPAPELLGGPSVFLFPPKPKDTLMI SR	/	Alkylation (iodoacetamide)(B223); Alkylation (iodoacetamide)(B229); Alkylation (iodoacetamide)(B232)	4150.0512	4150.0746	5.64	831.0200	48673	/
**42.339**	B(259-291)	TPEVTCVVVDVSHEDPEVKFNWYVDGVEVHNA K	/	Alkylation (iodoacetamide)(B264)	3796.8043	3796.8156	2.98	760.3709	2303480	2
**32.317**	B(278-291)	FNWYVDGVEVHNAK	/	/	1676.7947	1676.7998	3.02	559.9407	109616656	4

**10.375**	B(292-304)	TKPREEQYNSTYR	/	G0F(B300)	3115.3351	3115.3398	1.51	1039.4541	3095380	4
**11.942**	B(296-304)	EEQYNSTYR	/	G0F(B300)	2633.0386	2633.0413	1.01	1317.5279	1207182	2
**45.851**	B(305-320)	VVSVLTVLHQDWLNGK	/	/	1806.9992	1807.0044	2.88	603.3422	244945856	7
**42.955**	B(305-323)	VVSVLTVLHQDWLNGKEYK	/	/	2227.2001	2227.2065	2.85	557.8089	149652384	3
**12.673**	B(321-325)	EYKCK	/	Alkylation (iodoacetamide)(B324)	726.3371	726.3337	-4.58	364.1745	150224	/
**35.383**	B(326-329)	VSNK	/	/	446.2489	446.2500	2.40	447.2573	226940	1
**20.126**	B(326-337)	VSNKALPAPIEK	/	/	1265.7343	1265.7351	0.60	422.9193	97728	4
**21.950**	B(330-337)	ALPAPIEK	/	/	837.4960	837.4976	1.94	419.7560	114950848	2
**26.373**	B(330-341)	ALPAPIEKTISK	/	/	1266.7547	1266.7573	1.98	634.3848	67204	/
**24.406**	B(330-343)	ALPAPIEKTISKAK	/	/	1465.8868	1465.8880	0.80	489.6360	15570	/
**15.676**	B(338-341)	TISK	/	/	447.2693	447.2696	0.59	448.2768	49278	1
**24.175**	B(344-358)	GQPREPQVYTLPPSR	/	/	1723.9006	1723.9041	2.06	862.9594	546534	1
**24.434**	B(344-363)	GQPREPQVYTLPPSREEMTK	/	/	2342.1689	2342.1760	3.05	586.5515	4779444	3
**25.911**	B(348-358)	EPQVYTLPPSR	/	/	1285.6667	1285.6697	2.39	643.8423	4069773	3
**25.947**	B(348-363)	EPQVYTLPPSREEMTK	/	/	1903.9350	1903.9402	2.77	635.6540	24520314	2
**30.167**	B(359-373)	EEMTKNQVSLTCLVK	/	Alkylation (iodoacetamide)(B370)	1778.8907	1778.8938	1.76	593.9722	957084	2
**30.822**	B(364-373)	NQVSLTCLVK	/	Alkylation (iodoacetamide)(B370)	1160.6223	1160.6253	2.56	581.3200	178722048	3
**47.007**	B(359-395)	EEMTKNQVSLTCLVKGFYPSDIAVEWESNGQPE NNYK	/	Alkylation (iodoacetamide)(B370)	4304.0042	4304.0250	4.83	1077.0100	54235	/
**18.017**	B(374-412)	GFYPSDIAVEWESNGQPENNYKTTPPVLDSDGSF FLYSK	/	/	4269.9331	4269.9300	-0.74	1424.3200	71970	/
**40.401**	B(374-395)	GFYPSDIAVEWESNGQPENNYK	1*Deamidation(+0.984016)B387/B 392/B393	/	2544.1081	2544.1125	1.71	849.0500	136561	1
**39.620**	B(374-395)	GFYPSDIAVEWESNGQPENNYK	1*Deamidation(+0.984016)B387/B 392/B393	/	2544.1081	2544.1156	2.93	849.0500	305685	1

**39.749**	B(374-395)	GFYPSDIAVEWESNGQPENNYK	/	/	2543.1241	2543.1388	5.79	848.7200	7489774	1
**41.963**	B(396-412)	TTPPVLDSDGSFFLYSK	/	/	1872.9146	1872.9190	2.36	937.4664	105271704	3
**44.358**	B(396-417)	TTPPVLDSDGSFFLYSKLTVDK	/	/	2429.2366	2429.2409	1.75	810.7537	105661	/
**41.440**	B(396-419)	TTPPVLDSDGSFFLYSKLTVDKSR	/	/	2672.3698	2672.3765	2.52	669.1009	393700	/
**8.286**	B(413-417)	LTVDK	/	/	574.3326	574.3336	1.69	575.3410	20159270	2
**4.869**	B(413-419)	LTVDKSR	/	/	817.4658	817.4681	2.85	409.7413	1319388	2
**21.937**	B(418-419)	SR	/	/	261.1437	261.1445	2.90	262.1517	1320293	1
**41.882**	B(420-450)	WQQGNVFSCSVMHEALHNHYTQKSLSLSPGK	1*Oxidation (M)(+15.994915)B431	Alkylation (iodoacetamide) (B428)	3400.5717	3400.5859	4.17	851.1500	84029	/
**31.225**	B(420-442)	WQQGNVFSCSVMHEALHNHYTQK	/	Alkylation (iodoacetamide) (B428)	2800.2598	2800.2675	2.72	701.0700	37259899	1
**43.388**	B(418-442)	SRWQQGNVFSCSVMHEALHNHYTQK	1*Oxidation (M)(+15.994915)B431	Alkylation (iodoacetamide) (B428)	3002.3664	3002.3463	-6.69	751.5900	167793	/
**29.796**	B(418-442)	SRWQQGNVFSCSVMHEALHNHYTQK	/	Alkylation (iodoacetamide) (B428)	3043.3930	3043.4096	5.46	609.6900	456562	1
**24.265**	B(443-449)	SLSLSPG	Lys-loss 8	/	659.3490	659.3520	4.58	330.6800	34563660	1
**23.627**	B(443-450)	SLSLSPGK	/	/	787.4440	787.4439	-0.10	788.4500	24714	/

A: LC   B: HC

Table S7. The details of identified peptides digested from P4 in off-line 2D-LC-MS.

**RT**	**Seq Loc**	**Sequence**	**Pred Mods**	**Mods**	**Tgt Seq Mass**	**Mass**	**Diff (Bio, ppm)**	**m/z**	**Vol**	**MS/MS Count**
**28.948**	A(1-18)	DIQMTQSPSSLSASVGDR	/	/	1877.8789	1877.8808	1.01	939.9474	55509072	5
**33.540**	A(1-24)	DIQMTQSPSSLSASVGDRVTITCR	/	Alkylation (iodoacetamide)(A23)	2608.2585	2608.2632	1.78	870.4285	2572218	2
**12.545**	A(19-24)	VTITCR	/	Alkylation (iodoacetamide)(A23)	748.3902	748.3917	2.03	375.2031	53081600	2
**44.481**	A(25-61)	ASQDVNTAVAWYQQKPGKAPKLLIYSAS FLYSGVPSR	/	/	4040.1160	4040.1182	0.55	809.0300	501225	1
**23.178**	A(25-45)	ASQDVNTAVAWYQQKPGKAPK	1*Deamidation(+0.984016)A30	/	2287.1597	2287.1669	3.15	763.4000	219421	1
**24.197**	A(25-45)	ASQDVNTAVAWYQQKPGKAPK	/	/	2286.1757	2286.1796	1.70	1144.100 0	15356297	1
**24.805**	A(25-42)	ASQDVNTAVAWYQQKPGK	1*Deamidation(+0.984016)A30	/	1990.9749	1990.9777	1.41	664.6700	42719	1
**26.949**	A(25-42)	ASQDVNTAVAWYQQKPGK	1*Deamidation(+0.984016)A30	/	1990.9749	1990.9759	0.54	664.6700	139362	1
**28.003**	A(19-42)	VTITCRASQDVNTAVAWYQQKPGK	1*Deamidation(+0.984016)A30	/	2664.3330	2664.3162	-6.32	889.1100	30351	/
**45.379**	A(46-61)	LLIYSASFLYSGVPSR	/	/	1771.9509	1771.9530	1.20	886.9835	151762576	3
**44.465**	A(46-66)	LLIYSASFLYSGVPSRFSGSR	/	/	2306.2059	2306.2149	3.89	769.7455	3169276	2
**52.222**	A(46-103)	LLIYSASFLYSGVPSRFSGSRSGTDFTLTIS SLQPEDFATYYCQQHYTTPPTFGQGTK	/	Alkylation (iodoacetamide)(A88)	6475.1060	6475.1217	2.43	1080.197 1	267524	/
**45.724**	A(62-103)	FSGSRSGTDFTLTISSLQPEDFATYYCQQH YTTPPTFGQGTK	/	Alkylation (iodoacetamide)(A88)	4721.1657	4721.1633	-0.51	1181.298 2	235479	1
**47.893**	A(67-103)	SGTDFTLTISSLQPEDFATYYCQQHYTTPP TFGQGTK	/	Alkylation (iodoacetamide)(A88)	4186.9106	4186.9143	0.88	1047.737 0	455103	3
**46.872**	A(67-107)	SGTDFTLTISSLQPEDFATYYCQQHYTTPP TFGQGTKVEIK	/	Alkylation (iodoacetamide)(A88)	4656.2007	4656.2096	1.93	1165.058 9	528539	1

**44.882**	A(67-108)	SGTDFTLTISSLQPEDFATYYCQQHYTTPP TFGQGTKVEIKR	/	Alkylation (iodoacetamide)(A88)	4812.3018	4812.3092	1.54	963.4690	1234761	2
**5.814**	A(104-107)	VEIK	/	/	487.3006	487.3011	0.98	488.3084	2639735	1
**3.652**	A(104-108)	VEIKR	/	/	643.4017	643.4033	2.49	322.7091	17541204	2
**39.754**	A(108-126)	RTVAAPSVFIFPPSDEQLK	/	/	2101.1208	2101.1393	8.82	701.3874	9482554	3
**44.014**	A(109-126)	TVAAPSVFIFPPSDEQLK	/	/	1945.0197	1945.0240	2.24	649.3491	118150488	3
**41.293**	A(127-142)	SGTASVVCLLNNFYPR	/	Alkylation (iodoacetamide)(A134)	1797.8720	1797.8753	1.84	899.9448	1179098	2
**10.770**	A(146-149)	VQWK	/	/	559.3118	559.3131	2.25	560.3203	17272220	2
**26.769**	A(146-169)	VQWKVDNALQSGNSQESVTEQDSK	/	/	2676.2627	2676.2642	0.55	893.0957	102234	1
**36.442**	A(146-183)	VQWKVDNALQSGNSQESVTEQDSKDSTY SLSSTLTLSK	/	/	4160.0033	4160.0105	1.72	1041.007 9	548511	5
**18.027**	A(150-169)	VDNALQSGNSQESVTEQDSK	/	/	2134.9615	2134.9648	1.57	712.6622	16430320	4
**33.543**	A(150-183)	VDNALQSGNSQESVTEQDSKDSTYSLSST LTLSK	/	/	3618.7021	3618.7094	2.01	1207.243 0	2845519	3
**33.122**	A(150-188)	VDNALQSGNSQESVTEQDSKDSTYSLSST LTLSKADYEK	/	/	4224.9670	4224.9769	2.35	1057.248 2	60109	/
**33.546**	A(170-183)	DSTYSLSSTLTLSK	/	/	1501.7512	1501.7542	1.98	501.5920	178323	1
**21.158**	A(184-207)	ADYEKHKVYACEVTHQGLSSPVTK	/	Alkylation (iodoacetamide)(A194)	2746.3385	2746.3462	2.83	550.2766	705447	1
**19.395**	A(189-207)	HKVYACEVTHQGLSSPVTK	/	Alkylation (iodoacetamide)(A194)	2140.0735	2140.0792	2.64	536.0269	61894820	6
**23.701**	A(191-207)	VYACEVTHQGLSSPVTK	/	Alkylation (iodoacetamide)(A194)	1874.9197	1874.9225	1.54	625.9811	131761216	4
**2.310**	A(208-211)	SFNR	/	/	522.2550	522.2561	2.01	523.2634	2702486	2
**5.240**	A(208-214)	SFNRGEC	/	Alkylation (iodoacetamide)(A214)	868.3498	868.3516	2.12	435.1830	12109741	2
**33.857**	B(1-19)	EVQLVESGGGLVQPGGSLR	/	/	1880.9956	1880.9994	2.02	628.0074	116365400	5
**44.410**	B(1-38)	EVQLVESGGGLVQPGGSLRLSCAASGFNI KDTYIHWVR	/	Alkylation (iodoacetamide)(B22)	4100.0902	4100.0964	1.51	821.0269	858433	/

**25.388**	B(20-30)	LSCAASGFNIK	/	Alkylation (iodoacetamide)(B22)	1166.5754	1166.5777	1.98	584.2965	49387840	4
**36.914**	B(20-38)	LSCAASGFNIKDTYIHWVR	/	Alkylation (iodoacetamide)(B22)	2237.1052	2237.1089	1.67	560.2847	126755808	4
**27.872**	B(31-38)	DTYIHWVR	/	/	1088.5403	1088.5423	1.76	545.2782	29916174	3
**24.616**	B(39-50)	QAPGKGLEWVAR	/	/	1310.7095	1310.7129	2.59	437.9116	11275182	2
**26.880**	B(44-50)	GLEWVAR	/	/	829.4446	829.4461	1.79	415.7304	80375760	3
**17.991**	B(51-59)	IYPTNGYTR	/	/	1083.5349	1083.5365	1.47	542.7753	55255536	2
**4.464**	B(60-65)	YADSVK	/	/	681.3334	681.3348	2.15	341.6747	22069668	3
**17.960**	B(66-76)	GRFTISADTSK	/	/	1181.6041	1181.6062	1.84	591.8101	13036451	3
**31.924**	B(66-87)	GRFTISADTSKNTAYLQMNSLR	/	/	2473.2384	2473.2410	1.05	619.3176	2810497	2
**20.438**	B(68-76)	FTISADTSK	/	/	968.4815	968.4837	2.31	485.2498	67643488	2
**34.659**	B(68-87)	FTISADTSKNTAYLQMNSLR	/	/	2260.1158	2260.1198	1.78	754.3805	12176029	3
**28.516**	B(77-87)	NTAYLQMNSLR	/	/	1309.6449	1309.6470	1.60	655.8308	60745824	3
**18.502**	B(88-98)	AEDTAVYYCSR	/	Alkylation (iodoacetamide)(B96)	1333.5609	1333.5625	1.25	667.7885	27016396	3
**48.365**	B(99-124)	WGGDGFYAMDYWGQGTLVTVSSASTK	/	/	2783.2537	2783.2573	1.29	928.7597	7095488	4
**31.956**	B(125-136)	GPSVFPLAPSSK	/	/	1185.6394	1185.6418	2.01	593.8286	139482592	3
**55.988**	B(137-213)	STSGGTAALGCLVKDYFPEPVTVSWNSG ALTSGVHTFPAVLQSSGLYSLSSVVTVPSS SLGTQTYICNVNHKPSNTK	/	Alkylation (iodoacetamide)(B147); Alkylation (iodoacetamide)(B203)	8014.9674	8014.9843	2.11	1337.169 8	1224239	7
**54.188**	B(151-213)	DYFPEPVTVSWNSGALTSGVHTFPAVLQS SGLYSLSSVVTVPSSSLGTQTYICNVNHKP SNTK	/	Alkylation (iodoacetamide)(B203)	6712.3072	6712.3201	1.92	1343.469 3	42726404	15
**53.180**	B(151-216)	DYFPEPVTVSWNSGALTSGVHTFPAVLQS SGLYSLSSVVTVPSSSLGTQTYICNVNHKP SNTKVDK	/	Alkylation (iodoacetamide)(B203)	7054.4975	7054.5117	2.01	1176.758 5	9748924	10

**52.235**	B(151-217)	DYFPEPVTVSWNSGALTSGVHTFPAVLQS SGLYSLSSVVTVPSSSLGTQTYICNVNHKP SNTKVDKK	/	Alkylation (iodoacetamide)(B203)	7182.5925	7182.6095	2.37	1198.108 2	10083014	11
**33.558**	B(218-221)	VEPK	/	/	471.2693	471.2699	1.26	472.2770	56039	/
**41.609**	B(222-251)	SCDKTHTCPPCPAPELLGGPSVFLFPPKPK	/	Alkylation (iodoacetamide)(B223); Alkylation (iodoacetamide)(B229); Alkylation (iodoacetamide)(B232)	3333.6349	3333.6410	1.85	667.7357	105245984	6
**43.388**	B(226-251)	THTCPPCPAPELLGGPSVFLFPPKPK	/	Alkylation (iodoacetamide)(B229); Alkylation (iodoacetamide)(B232)	2843.4503	2843.4558	1.93	711.8712	65017504	4
**43.693**	B(222-258)	SCDKTHTCPPCPAPELLGGPSVFLFPPKPK DTLMISR	/	Alkylation (iodoacetamide)(B223); Alkylation (iodoacetamide)(B229); Alkylation (iodoacetamide)(B232)	4150.0512	831.02	-0.09	4150.050 9	42699	/
**43.518**	B(222-258)	SCDKTHTCPPCPAPELLGGPSVFLFPPKPK DTLMISR	/	Alkylation (iodoacetamide)(B223); Alkylation (iodoacetamide)(B229); Alkylation (iodoacetamide)(B232)	4150.0512	831.02	7.8	4150.083 6	170443	/
**18.456**	B(252-258)	DTLMISR	1*Oxidation (M)(+15.994915)B255	/	850.4219	851.43	5.8	850.4268	4419527	1
**21.94**	B(252-258)	DTLMISR	/	/	834.4269	835.44	2.37	834.4289	32281174	1
**33.551**	B(259-277)	TPEVTCVVVDVSHEDPEVK	/	Alkylation (iodoacetamide)(B264)	2138.0202	2138.0271	3.25	535.5140	1610372	1
**42.361**	B(259-291)	TPEVTCVVVDVSHEDPEVKFNWYVDGVE VHNAK	/	Alkylation (iodoacetamide)(B264)	3796.8043	3796.8106	1.65	760.3695	1990355	4
**47.687**	B(259-295)	TPEVTCVVVDVSHEDPEVKFNWYVDGVE VHNAKTKPR	/	Alkylation (iodoacetamide)(B264)	4279.1008	4279.1411	9.41	857.2386	149130	/
**32.346**	B(278-291)	FNWYVDGVEVHNAK	/	/	1676.7947	1676.7997	2.96	559.9401	108388352	3
**10.088**	B(292-304)	TKPREEQYNSTYR	/	G0F(B300)	3115.3351	3115.3407	1.78	1039.453 7	2968040	5

**11.808**	B(296-304)	EEQYNSTYR	/	G0F(B300)	2633.0386	2633.0424	1.43	1317.527<br>4	1122039	2
**45.885**	B(305-320)	VVSVLTVLHQDWLNGK	/	/	1806.9992	1807.0017	1.36	603.3413	226271200	7
**42.976**	B(305-323)	VVSVLTVLHQDWLNGKEYK	/	/	2227.2001	2227.2039	1.72	557.8084	170040368	4
**12.554**	B(321-325)	EYKCK	/	Alkylation (iodoacetamide)(B324)	726.3371	726.3338	-4.47	364.1741	118629	/
**35.424**	B(326-329)	VSNK	/	/	446.2489	446.2495	1.42	447.2568	218103	1
**20.142**	B(326-337)	VSNKALPAPIEK	/	/	1265.7343	1265.7382	3.06	422.9201	153235	3
**21.974**	B(330-337)	ALPAPIEK	/	/	837.4960	837.4973	1.54	419.7562	113270096	2
**15.602**	B(338-341)	TISK	/	/	447.2693	447.2688	-1.06	448.2762	41414	1
**21.352**	B(342-358)	AKGQPREPQVYTLPPSR	/	/	1923.0326	1923.0363	1.88	481.7665	1296692	5
**24.198**	B(344-358)	GQPREPQVYTLPPSR	/	/	1723.9006	1723.9029	1.33	862.9587	516519	1
**24.240**	B(344-363)	GQPREPQVYTLPPSREEMTK	/	/	2342.1689	2342.1711	0.96	1172.092<br>0	274736	/
**25.951**	B(348-358)	EPQVYTLPPSR	/	/	1285.6667	1285.6681	1.13	643.8414	2189049	2
**25.999**	B(348-363)	EPQVYTLPPSREEMTK	/	/	1903.9350	1903.9380	1.61	635.6532	17066650	2
**30.189**	B(359-373)	EEMTKNQVSLTCLVK	/	Alkylation (iodoacetamide)(B370)	1778.8907	1778.8928	1.18	593.9716	1221056	2
**30.847**	B(364-373)	NQVSLTCLVK	/	Alkylation (iodoacetamide)(B370)	1160.6223	1160.6240	1.42	581.3196	175862480	4
**39.657**	B(374-395)	GFYPSDIAVEWESNGQPENNYK	1*Deamidation(+0.984016)B387/B<br>392/B393	/	2544.1081	2544.117	3.48	1273.07	363529	1
**39.745**	B(374-395)	GFYPSDIAVEWESNGQPENNYK	/	/	2543.1241	2543.1275	1.32	1272.57	2294841	1
**41.980**	B(396-412)	TTPPVLDSDGSFFLYSK	/	/	1872.9146	1872.9173	1.49	937.4655	101826920	4
**41.447**	B(396-417)	TTPPVLDSDGSFFLYSKLTVDK	/	/	2429.2366	2429.2362	-0.15	810.7532	149046	/
**41.461**	B(396-419)	TTPPVLDSDGSFFLYSKLTVDKSR	/	/	2672.3698	2672.3723	0.93	669.1002	488643	/
**7.795**	B(413-417)	LTVDK	/	/	574.3326	574.3337	1.93	575.3410	17536106	2
**4.236**	B(413-419)	LTVDKSR	/	/	817.4658	817.4674	1.95	409.7410	1508240	3

**21.958**	B(418-419)	SR	/	/	261.1437	261.1441	1.45	262.1514	1235725	1
**26.29**	B(420-442)	WQQGNVFSCSVMHEALHNHYTQK	1*Oxidation (M)(+15.994915)B431	Alkylation (iodoacetamide) (B428)	2816.2548	705.07	1.73	2816.2596	1335907	1
**31.256**	B(420-442)	WQQGNVFSCSVMHEALHNHYTQK	/	Alkylation (iodoacetamide) (B428)	2800.2598	701.07	1.27	2800.2634	34549299	1
**29.734**	B(418-442)	SRWQQGNVFSCSVMHEALHNHYTQK	/	Alkylation (iodoacetamide) (B428)	3043.393	609.69	1.07	3043.3962	628902	1
**24.281**	B(443-449)	SLSLSPG	Lys-loss 8	/	659.349	659.3514	3.56	330.68	32332121	1
**19.215**	B(443-450)	SLSLSPGK	/	/	787.444	787.4462	2.86	788.45	1838365	1

A: LC B: HC

Table S8. The details of identified peptides digested from P5 in off-line 2D-LC-MS.

RT	Seq Loc	Sequence	Pred Mods	Mods	Tgt Seq Mass	Mass	Diff (Bio, ppm)	m/z	Vol	MS/MS Count
**28.959**	A(1-18)	DIQMTQSPSSLSASVGDR	/	/	1877.8789	1877.8826	1.94	939.9482	36717292	3
**33.445**	A(1-24)	DIQMTQSPSSLSASVGDRVTITCR	/	Alkylation (iodoacetamide)(A23)	2608.2585	2608.2635	1.90	1305.1382	9818738	2
**12.722**	A(19-24)	VTITCR	/	Alkylation (iodoacetamide)(A23)	748.3902	748.3918	2.23	375.2032	32015638	2
**44.461**	A(25-61)	ASQDVNTAVAWYQQKPGKAPKLLIYSASFL YSGVPSR	/	/	4040.1160	4040.1285	3.11	809.0300	83019	/
**24.681**	A(25-45)	ASQDVNTAVAWYQQKPGKAPK	/	/	2286.1757	2286.1733	-1.06	572.8000	41787	/
**23.163**	A(25-45)	ASQDVNTAVAWYQQKPGKAPK	1*Deamidation(+0.984016)A30	/	2287.1597	2287.1695	4.29	572.8000	740048	1
**25.156**	A(25-45)	ASQDVNTAVAWYQQKPGKAPK	1*Deamidation(+0.984016)A30	/	2287.1597	2287.1651	2.35	572.8000	1165164	2
**24.141**	A(25-45)	ASQDVNTAVAWYQQKPGKAPK	/	/	2286.1757	2286.1815	2.56	1144.1000	29454780	1
**26.933**	A(25-42)	ASQDVNTAVAWYQQKPGK	1*Deamidation(+0.984016)A30	/	1990.9749	1990.9786	1.88	996.5000	40450	/
**25.969**	A(25-42)	ASQDVNTAVAWYQQKPGK	/	/	1989.9908	1989.9956	2.39	664.3400	1005352	1
**45.351**	A(46-61)	LLIYSASFLYSGVPSR	/	/	1771.9509	1771.9535	1.48	886.9837	191471008	3
**44.434**	A(46-66)	LLIYSASFLYSGVPSRFSGSR	/	/	2306.2059	2306.2107	2.06	769.7442	16172850	3
**6.837**	A(62-66)	FSGSR	/	/	552.2656	552.2708	9.36	277.1426	428090	2
**47.929**	A(67-103)	SGTDFTLTISSLQPEDFATYYCQQHYTTPPTF GQGTK	/	Alkylation (iodoacetamide)(A88)	4186.9106	4186.9193	2.08	1047.7375	290506	1
**46.843**	A(67-107)	SGTDFTLTISSLQPEDFATYYCQQHYTTPPTF GQGTKVEIK	/	Alkylation (iodoacetamide)(A88)	4656.2007	4656.2085	1.68	1165.0581	6200497	3
**4.311**	A(104-108)	VEIKR	/	/	643.4017	643.4035	2.76	322.7090	14403915	3
**40.373**	A(108-126)	RTVAAPSVFIFPPSDEQLK	/	/	2101.1208	2101.1223	0.70	701.3813	1439949	2
**55.297**	A(108-142)	RTVAAPSVFIFPPSDEQLKSGTASVVCLLNN FYPR	/	Alkylation (iodoacetamide)(A134)	3879.9982	3880.0074	2.38	971.0079	737676	3

**44.014**	A(109-126)	TVAAPSVFIFPPSDEQLK	/	/	1945.0197	1945.0251	2.76	649.3496	156440768	3
**58.434**	A(109-142)	TVAAPSVFIFPPSDEQLKSGTASVVCLLNNF YPR	/	Alkylation (iodoacetamide)(A134)	3723.8971	3723.9008	1.00	931.9826	9097575	5
**12.506**	A(143-149)	EAKVQWK	/	/	887.4865	887.4857	-0.89	444.7507	48400	1
**11.018**	A(146-149)	VQWK	/	/	559.3118	559.3131	2.21	560.3202	14674306	2
**36.381**	A(146-183)	VQWKVDNALQSGNSQESVTEQDSKDSTYS LSSTLTLSK	/	/	4160.0033	4160.0137	2.49	1041.0107	17783510	5
**18.046**	A(150-169)	VDNALQSGNSQESVTEQDSK	/	/	2134.9615	2134.9650	1.68	712.6624	8862727	4
**33.584**	A(170-183)	DSTYSLSSTLTLSK	/	/	1501.7512	1501.7536	1.59	501.5915	161819	1
**20.966**	A(184-207)	ADYEKHKVYACEVTHQGLSSPVTK	/	Alkylation (iodoacetamide)(A194)	2746.3385	2746.3463	2.84	550.2768	53862680	7
**19.372**	A(189-207)	HKVYACEVTHQGLSSPVTK	/	Alkylation (iodoacetamide)(A194)	2140.0735	2140.0789	2.49	536.0269	80805336	5
**23.701**	A(191-207)	VYACEVTHQGLSSPVTK	/	Alkylation (iodoacetamide)(A194)	1874.9197	1874.9244	2.52	625.9821	109699904	4
**2.856**	A(208-211)	SFNR	/	/	522.2550	522.2561	1.94	523.2633	2211712	1
**5.822**	A(208-214)	SFNRGEC	/	Alkylation (iodoacetamide)(A214)	868.3498	868.3507	1.14	435.1824	22330394	2
**33.816**	B(1-19)	EVQLVESGGGLVQPGGSLR	/	/	1880.9956	1880.9995	2.09	941.5066	145092656	4
**44.367**	B(1-38)	EVQLVESGGGLVQPGGSLRLSCAASGFNIK DTYIHWVR	/	Alkylation (iodoacetamide)(B22)	4100.0902	4100.0949	1.15	821.0265	1800520	2
**25.420**	B(20-30)	LSCAASGFNIK	/	Alkylation (iodoacetamide)(B22)	1166.5754	1166.5790	3.13	584.2975	17401190	3
**36.824**	B(20-38)	LSCAASGFNIKDTYIHWVR	/	Alkylation (iodoacetamide)(B22)	2237.1052	2237.1108	2.51	560.2853	236888944	4
**27.895**	B(31-38)	DTYIHWVR	/	/	1088.5403	1088.5429	2.39	545.2786	10880870	3
**20.146**	B(39-43)	QAPGK	/	/	499.2754	499.2762	1.49	500.2831	148685	1
**24.474**	B(39-50)	QAPGKGLEWVAR	/	/	1310.7095	1310.7135	3.03	437.9121	102379968	3
**26.908**	B(44-50)	GLEWVAR	/	/	829.4446	829.4467	2.48	415.7307	42637168	2
**17.990**	B(51-59)	IYPTNGYTR	/	/	1083.5349	1083.5370	1.96	542.7758	62755344	2
**24.291**	B(60-65)	YADSVK	/	/	681.3334	681.3319	-2.11	682.3390	19272680	1

**3.576**	B(60-67)	YADSVKGR	/	/	894.4559	894.4580	2.28	448.2363	5805223	4
**17.959**	B(66-76)	GRFTISADTSK	/	/	1181.6041	1181.6069	2.39	591.8105	10251004	3
**20.472**	B(68-76)	FTISADTSK	/	/	968.4815	968.4835	2.12	485.2489	36337628	2
**37.184**	B(68-98)	FTISADTSKNTAYLQMNSLRAEDTAVYYCS R	/	Alkylation (iodoacetamide)(B96)	3575.6661	3575.6742	2.26	894.9258	5913284	5
**28.546**	B(77-87)	NTAYLQMNSLR	/	/	1309.6449	1309.6473	1.87	655.8310	34614236	3
**18.511**	B(88-98)	AEDTAVYYCSR	/	Alkylation (iodoacetamide)(B96)	1333.5609	1333.5634	1.90	667.7891	29363370	3
**48.352**	B(99-124)	WGGDGFYAMDYWGQGTLVTVSSASTK	/	/	2783.2537	2783.2556	0.65	928.7593	7577310	3
**52.964**	B(99-136)	WGGDGFYAMDYWGQGTLVTVSSASTKGPS VFPLAPSSK	/	/	3950.8826	3950.8885	1.51	988.7298	617414	3
**28.006**	B(137-150)	STSGGTAALGCLVK	/	Alkylation (iodoacetamide)(B147)	1320.6708	1320.6730	1.70	661.3437	125340824	3
**55.923**	B(137-213)	STSGGTAALGCLVKDYFPEPVTVSWNSGAL TSGVHTFPAVLQSSGLYSLSSVVTVPSSSLG TQTYICNVNHKPSNTK	/	Alkylation (iodoacetamide)(B147); Alkylation (iodoacetamide)(B203)	8014.9674	8014.9816	1.77	1336.8376	6906480	11
**55.149**	B(137-216)	STSGGTAALGCLVKDYFPEPVTVSWNSGAL TSGVHTFPAVLQSSGLYSLSSVVTVPSSSLG TQTYICNVNHKPSNTKVDK	/	Alkylation (iodoacetamide)(B147); Alkylation (iodoacetamide)(B203)	8357.1577	8357.1889	3.73	1194.8884	7560924	13
**54.247**	B(151-213)	DYFPEPVTVSWNSGALTSGVHTFPAVLQSS GLYSLSSVVTVPSSSLGTQTYICNVNHKPSN TK	/	Alkylation (iodoacetamide)(B203)	6712.3072	6712.3190	1.76	1343.4690	17346462	9
**53.146**	B(151-216)	DYFPEPVTVSWNSGALTSGVHTFPAVLQSS GLYSLSSVVTVPSSSLGTQTYICNVNHKPSN TKVDK	/	Alkylation (iodoacetamide)(B203)	7054.4975	7054.5218	3.44	1176.7579	18903172	14
**52.053**	B(151-217)	DYFPEPVTVSWNSGALTSGVHTFPAVLQSS GLYSLSSVVTVPSSSLGTQTYICNVNHKPSN TKVDKK	/	Alkylation (iodoacetamide)(B203)	7182.5925	7182.6142	3.02	1027.0938	3584679	/

**8.348**	B(214-216)	VDK	/	/	360.2009	360.2017	2.17	361.2090	5698972	1
**41.536**	B(222-251)	SCDKTHTCPPCPAPELLGGPSVFLFPPKPK	/	Alkylation (iodoacetamide)(B223);<br>Alkylation (iodoacetamide)(B229);<br>Alkylation (iodoacetamide)(B232)	3333.6349	3333.6519	5.12	667.7359	142348080	6
**43.399**	B(226-251)	THTCPPCPAPELLGGPSVFLFPPKPK	/	Alkylation (iodoacetamide)(B229);<br>Alkylation (iodoacetamide)(B232)	2843.4503	2843.4570	2.37	711.8718	22455618	3
**45.453**	B(252-291)	DTLMISRTPEVTCVVVDVSHEDPEVKFNWY<br>VDGVEVHNAK	/	Alkylation (iodoacetamide)(B264)	4613.2207	4613.2141	-1.43	923.6500	30801	/
**37.874**	B(252-277)	DTLMISRTPEVTCVVVDVSHEDPEVK	1*Oxidation (M)(+15.994915)B255	Alkylation (iodoacetamide)(B264)	2970.4314	2970.4333	0.62	743.6200	25802	1
**39.690**	B(252-277)	DTLMISRTPEVTCVVVDVSHEDPEVK	/	Alkylation (iodoacetamide)(B264)	2954.4365	2954.4405	1.33	985.8200	63951	/
**18.465**	B(252-258)	DTLMISR	1*Oxidation (M)(+15.994915)B255	/	850.4219	850.4241	2.63	851.4300	3454474	1
**21.941**	B(252-258)	DTLMISR	/	/	834.4269	834.4293	2.88	835.4400	31686460	1
**45.143**	B(226-258)	THTCPPCPAPELLGGPSVFLFPPKPKDTLMIS<br>R	/	Alkylation (iodoacetamide)(B229);<br>Alkylation (iodoacetamide)(B232)	3659.8666	3659.8732	1.79	915.9800	51443	1
**45.314**	B(226-258)	THTCPPCPAPELLGGPSVFLFPPKPKDTLMIS<br>R	/	Alkylation (iodoacetamide)(B229);<br>Alkylation (iodoacetamide)(B232)	3602.8452	3602.8322	-3.61	1202.2900	102068	/
**42.156**	B(222-258)	SCDKTHTCPPCPAPELLGGPSVFLFPPKPKD<br>TLMISR	1*Oxidation (M)(+15.994915)B255	Alkylation (iodoacetamide)(B223);<br>Alkylation (iodoacetamide)(B229);<br>Alkylation (iodoacetamide)(B232)	4166.0461	4166.0447	-0.34	695.3500	310782	1
**43.717**	B(222-258)	SCDKTHTCPPCPAPELLGGPSVFLFPPKPKD<br>TLMISR	/	Alkylation (iodoacetamide)(B223);<br>Alkylation (iodoacetamide)(B229);<br>Alkylation (iodoacetamide)(B232)	4150.0512	4150.0625	2.71	831.0200	1109251	/
**43.410**	B(222-258)	SCDKTHTCPPCPAPELLGGPSVFLFPPKPKD<br>TLMISR	/	Alkylation (iodoacetamide)(B223);<br>Alkylation (iodoacetamide)(B229);<br>Alkylation (iodoacetamide)(B232)	4150.0512	4150.0634	2.93	831.0200	6744507	1
**27.375**	B(278-295)	FNWYVDGVEVHNAKTKPR	/	/	2159.0912	2159.0934	1.00	540.7801	52332	2

**10.382**	B(292-304)	TKPREEQYNSTYR	/	G0F(B300)	3115.3351	3115.3413	1.98	1039.4537	5382664	4
**11.965**	B(296-304)	EEQYNSTYR	/	G0F(B300)	2633.0386	2633.0385	-0.03	878.6866	510626	2
**45.840**	B(296-320)	EEQYNSTYRVVSVLTVLHQDWLNGK	/	G0F(B300)	4422.0273	4422.0406	3.01	1106.5168	199134	/
**43.908**	B(296-323)	EEQYNSTYRVVSVLTVLHQDWLNGKEYK	/	G0F(B300)	4842.2282	4842.2380	2.03	969.4553	1474179	/
**45.952**	B(305-320)	VVSVLTVLHQDWLNGK	/	/	1806.9992	1807.0024	1.74	603.3414	95505112	5
**42.879**	B(305-323)	VVSVLTVLHQDWLNGKEYK	/	/	2227.2001	2227.2046	2.03	557.8085	336376800	4
**40.352**	B(305-325)	VVSVLTVLHQDWLNGKEYKCK	/	Alkylation (iodoacetamide)(B324)	2515.3257	2515.3306	1.93	504.0743	129578	/
**18.735**	B(324-337)	CKVSNKALPAPIEK	/	Alkylation (iodoacetamide)(B324)	1553.8600	1553.8645	2.95	518.9618	495010	3
**20.146**	B(326-337)	VSNKALPAPIEK	/	/	1265.7343	1265.7380	2.89	422.9203	3353438	2
**21.949**	B(330-337)	ALPAPIEK	/	/	837.4960	837.4981	2.45	419.7564	136159328	2
**26.409**	B(330-341)	ALPAPIEKTISK	/	/	1266.7547	1266.7572	1.94	423.2602	191315	1
**21.330**	B(342-358)	AKGQPREPQVYTLPPSR	/	/	1923.0326	1923.0377	2.62	481.7665	1587911	1
**23.716**	B(344-358)	GQPREPQVYTLPPSR	/	/	1723.9006	1723.9029	1.37	862.9587	357265	1
**24.272**	B(344-363)	GQPREPQVYTLPPSREEMTK	/	/	2342.1689	2342.1729	1.69	781.7316	7117696	2
**25.436**	B(348-358)	EPQVYTLPPSR	/	/	1285.6667	1285.6691	1.87	643.8419	1425554	2
**26.000**	B(348-363)	EPQVYTLPPSREEMTK	/	/	1903.9350	1903.9395	2.37	635.6538	12967899	3
**36.086**	B(348-373)	EPQVYTLPPSREEMTKNQVSLTCLVK	/	Alkylation (iodoacetamide)(B370)	3046.5468	3046.5539	2.35	762.6456	11982187	1
**30.848**	B(364-373)	NQVSLTCLVK	/	Alkylation (iodoacetamide)(B370)	1160.6223	1160.6257	2.88	581.3207	175829376	3
**40.373**	B(374-395)	GFYPSDIAVEWESNGQPENNYK	1*Deamidation(+0.984016)B387/B392/B393	/	2544.1081	2544.1129	1.88	1273.0600	138610	1
**39.612**	B(374-395)	GFYPSDIAVEWESNGQPENNYK	1*Deamidation(+0.984016)B387/B392/B393	/	2544.1081	2544.1334	9.93	1273.0800	237127	2
**39.761**	B(374-395)	GFYPSDIAVEWESNGQPENNYK	/	/	2543.1241	2543.1392	5.93	848.7200	4038440	1
**41.947**	B(396-412)	TTPPVLDSDGSFFLYSK	/	/	1872.9146	1872.9173	1.44	937.4655	103766016	3
**44.352**	B(396-417)	TTPPVLDSDGSFFLYSKLTVDK	/	/	2429.2366	2429.2367	0.04	810.7527	426064	/

**41.411**	B(396-419)	TTPPVLDSDGSFFLYSKLTVDKSR	/	/	2672.3698	2672.3757	2.21	669.1007	1173513	/
**8.343**	B(413-417)	LTVDK	/	/	574.3326	574.3337	1.92	575.3409	13884528	2
**31.851**	B(413-417)	LTVDK	/	/	574.3326	574.3362	6.30	575.3432	50132	/
**4.881**	B(413-419)	LTVDKSR	/	/	817.4658	817.4675	2.11	409.7410	9231000	2
**21.953**	B(418-419)	SR	/	/	261.1437	261.1442	1.98	262.1515	1222231	1
**26.288**	B(420-442)	WQQGNVFSCSVMHEALHNHYTQK	1*Oxidation (M)(+15.994915)B431	Alkylation (iodoacetamide) (B428)	2816.2548	2816.2614	2.36	705.0700	923618	1
**31.215**	B(420-442)	WQQGNVFSCSVMHEALHNHYTQK	/	Alkylation (iodoacetamide) (B428)	2800.2598	2800.2655	2.03	1401.1400	42594201	1
**30.303**	B(418-450)	SRWQQGNVFSCSVMHEALHNHYTQKSLSL SPGK	1*Oxidation (M)(+15.994915)B431	/	3771.7998	3771.8107	2.89	755.5700	56945	/
**29.689**	B(418-442)	SRWQQGNVFSCSVMHEALHNHYTQK	/	Alkylation (iodoacetamide) (B428)	3043.3930	3043.4007	2.53	609.6900	2105659	1
**32.250**	B(413-442)	LTVDKSRWQQGNVFSCSVMHEALHNHYTQ K	1*Oxidation (M)(+15.994915)B431	/	3430.5935	3430.5799	-3.98	1716.3000	38636	/
**24.293**	B(443-449)	SLSLSPG	Lys-loss K450	/	659.3490	659.3512	3.26	660.3600	25251678	1
**19.186**	B(443-450)	SLSLSPGK	/	/	787.4440	787.4479	5.02	788.4500	26794414	1

A: LC B: HC

Table S9. The details of identified peptides digested from P6 in off-line 2D-LC-MS.

RT	Seq Loc	Sequence	Pred Mods	Mods	Tgt Seq Mass	Mass	Diff (Bio, ppm)	m/z	Vol	MS/MS Count
**28.976**	A(1-18)	DIQMTQSPSSLSASVGDR	/	/	1877.8789	1877.8829	2.10	939.9484	42320792	4
**12.720**	A(19-24)	VTITCR	/	Alkylation (iodoacetamide)(A23)	748.3902	748.3918	2.25	375.2032	40174272	3
**44.516**	A(25-61)	ASQDVNTAVAWYQQKPGKAPKLLIYSASFL YSGVPSR	/	/	4040.1160	4040.1369	5.17	809.0400	220982	1
**23.185**	A(25-45)	ASQDVNTAVAWYQQKPGKAPK	1*Deamidation(+0.984016)A30	/	2287.1597	2287.1619	0.96	763.3900	299868	1
**25.190**	A(25-45)	ASQDVNTAVAWYQQKPGKAPK	1*Deamidation(+0.984016)A30	/	2287.1597	2287.1668	3.12	572.8000	692551	1
**24.225**	A(25-45)	ASQDVNTAVAWYQQKPGKAPK	/	/	2286.1757	2286.1814	2.48	1144.1000	12813451	1
**24.816**	A(25-42)	ASQDVNTAVAWYQQKPGK	1*Deamidation(+0.984016)A30	/	1990.9749	1990.9709	-1.98	664.6600	44544	1
**26.975**	A(25-42)	ASQDVNTAVAWYQQKPGK	1*Deamidation(+0.984016)A30	/	1990.9749	1990.9805	2.81	664.6700	127566	1
**25.988**	A(25-42)	ASQDVNTAVAWYQQKPGK	/	/	1989.9908	1989.9959	2.56	996.0000	2272877	1
**45.424**	A(46-61)	LLIYSASFLYSGVPSR	/	/	1771.9509	1771.9553	2.47	886.9844	116137480	3
**44.495**	A(46-66)	LLIYSASFLYSGVPSRFSGSR	/	/	2306.2059	2306.2182	5.31	769.7466	3189046	3
**44.915**	A(67-108)	SGTDFTLTISSLQPEDFATYYCQQHYTTPPTF GQGTKVEIKR	/	Alkylation (iodoacetamide)(A88)	4812.3018	4812.3148	2.71	963.4699	1449369	2
**4.319**	A(104-108)	VEIKR	/	/	643.4017	643.4034	2.66	322.7089	14125546	3
**44.065**	A(109-126)	TVAAPSVFIFPPSDEQLK	/	/	1945.0197	1945.0259	3.17	649.3492	98150288	2
**58.464**	A(109-142)	TVAAPSVFIFPPSDEQLKSGTASVVCLLNNF YPR	/	Alkylation (iodoacetamide)(A134)	3723.8971	3723.9066	2.55	931.9847	500135	2
**7.903**	A(143-145)	EAK	/	/	346.1852	346.1852	-0.09	347.1929	13313	1
**11.045**	A(146-149)	VQWK	/	/	559.3118	559.3131	2.19	560.3203	13245372	2
**26.767**	A(146-169)	VQWKVDNALQSGNSQESVTEQDSK	/	/	2676.2627	2676.2672	1.67	893.0940	127396	1
**33.087**	A(146-169)	VQWKVDNALQSGNSQESVTEQDSK	/	/	2676.2627	2676.2752	4.67	670.0786	208926	/

**36.446**	A(146-183)	VQWKVDNALQSGNSQESVTEQDSKDSTYS LSSTLTLSK	/	/	4160.0033	4160.0140	2.56	1041.0115	423824	3
**18.035**	A(150-169)	VDNALQSGNSQESVTEQDSK	/	/	2134.9615	2134.9669	2.56	712.6630	13084703	4
**21.158**	A(184-207)	ADYEKHKVYACEVTHQGLSSPVTK	/	Alkylation (iodoacetamide)(A194)	2746.3385	2746.3480	3.47	550.2765	1017273	4
**19.402**	A(189-207)	HKVYACEVTHQGLSSPVTK	/	Alkylation (iodoacetamide)(A194)	2140.0735	2140.0803	3.18	536.0276	53806916	7
**23.716**	A(191-207)	VYACEVTHQGLSSPVTK	/	Alkylation (iodoacetamide)(A194)	1874.9197	1874.9243	2.48	625.9822	106894136	4
**2.841**	A(208-211)	SFNR	/	/	522.2550	522.2561	2.04	523.2634	3329794	2
**5.876**	A(208-214)	SFNRGEC	/	Alkylation (iodoacetamide)(A214)	868.3498	868.3517	2.22	435.1831	10083183	4
**33.885**	B(1-19)	EVQLVESGGGLVQPGGSLR	/	/	1880.9956	1881.0009	2.85	628.0079	98084528	3
**44.444**	B(1-38)	EVQLVESGGGLVQPGGSLRLSCAASGFNIK DTYIHWVR	/	Alkylation (iodoacetamide)(B22)	4100.0902	4100.1089	4.56	821.0296	493980	/
**25.423**	B(20-30)	LSCAASGFNIK	/	Alkylation (iodoacetamide)(B22)	1166.5754	1166.5765	0.94	584.2950	33256346	4
**36.920**	B(20-38)	LSCAASGFNIKDTYIHWVR	/	Alkylation (iodoacetamide)(B22)	2237.1052	2237.1131	3.54	560.2858	113440728	3
**27.927**	B(31-38)	DTYIHWVR	/	/	1088.5403	1088.5441	3.49	545.2792	21531972	3
**24.610**	B(39-50)	QAPGKGLEWVAR	/	/	1310.7095	1310.7139	3.36	437.9120	15318015	2
**26.919**	B(44-50)	GLEWVAR	/	/	829.4446	829.4467	2.49	415.7304	63857116	2
**18.015**	B(51-59)	IYPTNGYTR	/	/	1083.5349	1083.5376	2.51	542.7761	46578308	3
**5.025**	B(60-65)	YADSVK	/	/	681.3334	681.3346	1.80	341.6745	17412410	3
**3.593**	B(60-67)	YADSVKGR	/	/	894.4559	894.4574	1.67	448.2360	579739	3
**17.994**	B(66-76)	GRFTISADTSK	/	/	1181.6041	1181.6073	2.78	591.8108	12386080	3
**31.913**	B(66-87)	GRFTISADTSKNTAYLQMNSLR	/	/	2473.2384	2473.2456	2.92	619.3189	3805517	2
**20.464**	B(68-76)	FTISADTSK	/	/	968.4815	968.4840	2.60	485.2494	51635984	2
**34.665**	B(68-87)	FTISADTSKNTAYLQMNSLR	/	/	2260.1158	2260.1224	2.94	754.3814	13745631	3
**37.205**	B(68-98)	FTISADTSKNTAYLQMNSLRAEDTAVYYCS R	/	Alkylation (iodoacetamide)(B96)	3575.6661	3575.6773	3.12	894.9263	479068	2

**28.559**	B(77-87)	NTAYLQMNSLR	/	/	1309.6449	1309.6487	2.89	655.8318	47770372	3
**18.533**	B(88-98)	AEDTAVYYCSR	/	Alkylation (iodoacetamide)(B96)	1333.5609	1333.5634	1.87	667.7885	19848936	3
**48.378**	B(99-124)	WGGDGFYAMDYWGQGTLVTVSSASTK	/	/	2783.2537	2783.2608	2.53	928.7609	7031534	5
**52.277**	B(99-136)	WGGDGFYAMDYWGQGTLVTVSSASTKGPS VFPLAPSSK	/	/	3950.8826	3950.8910	2.14	988.7296	943121	3
**31.974**	B(125-136)	GPSVFPLAPSSK	/	/	1185.6394	1185.6423	2.46	593.8286	119908560	3
**28.055**	B(137-150)	STSGGTAALGCLVK	/	Alkylation (iodoacetamide)(B147)	1320.6708	1320.6747	2.95	661.3447	113734872	3
**55.992**	B(137-213)	STSGGTAALGCLVKDYFPEPVTVSWNSGAL TSGVHTFPAVLQSSGLYSLSSVVTVPSSSLG TQTYICNVNHKPSNTK	/	Alkylation (iodoacetamide)(B147); Alkylation (iodoacetamide)(B203)	8014.9674	8014.9974	3.75	1337.1742	487694	5
**54.244**	B(151-213)	DYFPEPVTVSWNSGALTSGVHTFPAVLQSS GLYSLSSVVTVPSSSLGTQTYICNVNHKPSN TK	/	Alkylation (iodoacetamide)(B203)	6712.3072	6712.3258	2.76	1343.4714	26247100	12
**53.214**	B(151-216)	DYFPEPVTVSWNSGALTSGVHTFPAVLQSS GLYSLSSVVTVPSSSLGTQTYICNVNHKPSN TKVDK	/	Alkylation (iodoacetamide)(B203)	7054.4975	7054.5217	3.43	882.8252	5191900	4
**52.253**	B(151-217)	DYFPEPVTVSWNSGALTSGVHTFPAVLQSS GLYSLSSVVTVPSSSLGTQTYICNVNHKPSN TKVDKK	/	Alkylation (iodoacetamide)(B203)	7182.5925	7182.6148	3.11	1198.1091	11726106	9
**41.651**	B(222-251)	SCDKTHTCPPCPAPELLGGPSVFLFPPKPK	/	Alkylation (iodoacetamide)(B223); Alkylation (iodoacetamide)(B229); Alkylation (iodoacetamide)(B232)	3333.6349	3333.6480	3.95	667.7374	85032680	5
**43.432**	B(226-251)	THTCPPCPAPELLGGPSVFLFPPKPK	/	Alkylation (iodoacetamide)(B229); Alkylation (iodoacetamide)(B232)	2843.4503	2843.4594	3.20	711.8723	37498580	4
**41.846**	B(252-291)	DTLMISRTPEVTCVVVDVSHEDPEVKFNWY VDGVEVHNAK	/	Alkylation (iodoacetamide)(B264)	4613.2207	4613.2028	-3.88	770.0400	20646	/

**18.492**	B(252-258)	DTLMISR	1*Oxidation (M)(+15.994915)B255	/	850.4219	850.4249	3.56	426.2200	1875806	1
**21.975**	B(252-258)	DTLMISR	/	/	834.4269	834.4302	3.95	835.4400	27549263	1
**31.868**	B(226-258)	THTCPPCPAPELLGGPSVFLFPPKPKDTLMIS R	/	/	3545.8237	3545.7972	-7.47	592.1400	243237	/
**43.756**	B(222-258)	SCDKTHTCPPCPAPELLGGPSVFLFPPKPKD TLMISR	/	Alkylation (iodoacetamide)(B223); Alkylation (iodoacetamide)(B229); Alkylation (iodoacetamide)(B232)	4150.0512	4150.0580	1.63	831.0200	30261	/
**43.571**	B(222-258)	SCDKTHTCPPCPAPELLGGPSVFLFPPKPKD TLMISR	/	Alkylation (iodoacetamide)(B223); Alkylation (iodoacetamide)(B229); Alkylation (iodoacetamide)(B232)	4150.0512	4150.0604	2.21	831.0200	90789	/
**32.375**	B(278-291)	FNWYVDGVEVHNAK	/	/	1676.7947	1676.7991	2.60	559.9401	87128608	3
**10.408**	B(292-304)	TKPREEQYNSTYR	/	G0F(B300)	3115.3351	3115.3396	1.42	1039.4529	2228849	4
**11.964**	B(296-304)	EEQYNSTYR	/	G0F(B300)	2633.0386	2633.0422	1.36	1317.5270	715397	3
**45.941**	B(305-320)	VVSVLTVLHQDWLNGK	/	/	1806.9992	1807.0049	3.16	603.3422	172684624	7
**42.998**	B(305-323)	VVSVLTVLHQDWLNGKEYK	/	/	2227.2001	2227.2079	3.49	557.8093	173268688	4
**40.282**	B(305-325)	VVSVLTVLHQDWLNGKEYKCK	/	Alkylation (iodoacetamide)(B324)	2515.3257	2515.3382	4.96	629.8416	232527	2
**35.444**	B(326-329)	VSNK	/	/	446.2489	446.2503	3.19	447.2575	241116	1
**20.161**	B(326-337)	VSNKALPAPIEK	/	/	1265.7343	1265.7368	1.95	422.9198	111930	3
**21.997**	B(330-337)	ALPAPIEK	/	/	837.4960	837.4988	3.36	419.7572	98899616	2
**15.682**	B(338-341)	TISK	/	/	447.2693	447.2694	0.15	448.2767	28668	1
**24.290**	B(344-363)	GQPREPQVYTLPPSREEMTK	/	/	2342.1689	2342.1736	2.02	781.7314	1868486	2
**25.460**	B(348-358)	EPQVYTLPPSR	/	/	1285.6667	1285.6693	2.06	643.8418	3697260	2
**36.120**	B(348-373)	EPQVYTLPPSREEMTKNQVSLTCLVK	/	Alkylation (iodoacetamide)(B370)	3046.5468	3046.5546	2.57	762.6455	1087248	3
**30.211**	B(359-373)	EEMTKNQVSLTCLVK	/	Alkylation (iodoacetamide)(B370)	1778.8907	1778.8948	2.35	593.9724	1324184	2
**30.885**	B(364-373)	NQVSLTCLVK	/	Alkylation (iodoacetamide)(B370)	1160.6223	1160.6257	2.85	581.3203	150435440	3

**40.391**	B(374-395)	GFYPSDIAVEWESNGQPENNYK	1*Deamidation(+0.984016)B387/B392/B393	/	2544.1081	2544.1058	-0.89	1273.0600	188706	1
**39.669**	B(374-395)	GFYPSDIAVEWESNGQPENNYK	1*Deamidation(+0.984016)B387/B392/B393	/	2544.1081	2544.1222	5.54	849.0500	440986	1
**39.907**	B(374-395)	GFYPSDIAVEWESNGQPENNYK	/	/	2543.1241	2543.1463	8.74	1272.5800	581109	1
**39.776**	B(374-395)	GFYPSDIAVEWESNGQPENNYK	/	/	2543.1241	2543.1471	9.05	848.7200	3313193	1
**42.010**	B(396-412)	TTPPVLDSDGSFFLYSK	/	/	1872.9146	1872.9200	2.91	937.4670	82521984	3
**41.485**	B(396-417)	TTPPVLDSDGSFFLYSKLTVDK	/	/	2429.2366	2429.2431	2.69	810.7548	127811	/
**41.484**	B(396-419)	TTPPVLDSDGSFFLYSKLTVDKSR	/	/	2672.3698	2672.3746	1.82	891.7996	73318	/
**8.346**	B(413-417)	LTVDK	/	/	574.3326	574.3340	2.41	575.3411	13711573	3
**4.932**	B(413-419)	LTVDKSR	/	/	817.4658	817.4680	2.67	409.7413	1398723	2
**21.981**	B(418-419)	SR	/	/	261.1437	261.1444	2.62	262.1517	1163101	1
**41.602**	B(420-450)	WQQGNVFSCSVMHEALHNHYTQKSLSLSPGK	1*Oxidation (M)(+15.994915)B431	/	3400.5717	3400.5764	1.38	1134.5300	36143	/
**41.936**	B(420-450)	WQQGNVFSCSVMHEALHNHYTQKSLSLSPGK	1*Oxidation (M)(+15.994915)B431	/	3400.5717	3400.5924	6.08	851.1600	69153	/
**26.535**	B(420-442)	WQQGNVFSCSVMHEALHNHYTQK	1*Oxidation (M)(+15.994915)B431	Alkylation (iodoacetamide) (B428)	2816.2548	2816.2638	3.22	705.0700	399828	1
**31.282**	B(420-442)	WQQGNVFSCSVMHEALHNHYTQK	/	Alkylation (iodoacetamide) (B428)	2800.2598	2800.2673	2.67	561.0600	29238406	1
**29.802**	B(418-442)	SRWQQGNVFSCSVMHEALHNHYTQK	/	Alkylation (iodoacetamide) (B428)	3043.3930	3043.4033	3.39	609.6900	610603	1
**24.309**	B(443-449)	SLSLSPG	Lys-loss K450	/	659.3490	659.3514	3.66	660.3600	27396934	1
**19.266**	B(443-450)	SLSLSPGK	/	/	787.4440	787.4471	3.99	394.7300	2020033	1

A: LC B: HC

Table S10. The fragment ions of corresponding peptide with HC-N387 deamidation in P1.

Ion	Sequence	m/z (prod.)	m/z	Diff (Bio, ppm)	Abund
**b11**	GFYPSDIAVEW	1265.5837	1265.5684	-12.1	100
**y3**	YSK	397.2082	397.2106	6.1	107.6
**y7**	SFFLYSK	891.4611	891.4619	1	73.3
**y11**	DSDGSFFLYSK	1265.5685	1265.5684	-0.1	100
**y15**	PPVLDSDGSFFLYSK	836.4169	836.4093	-9.1	165.8
**y23**	PENNYKTTPPVLDSDGSFFLYSK	1310.6263	1310.618	-6.3	118.7
**y30**	EWESNGQPENNYKTTPPVLDSDGSFFLYSK	1150.8598	1150.8636	3.3	102.72
**Y**		136.0757	136.0755	-1.4	329.5

Table S11. The fragment ions of corresponding peptide without HC-N387 deamidation in P1.

Ion	Sequence	m/z (prod.)	m/z	Diff (Bio, ppm)	Abund
**b8**	GFYPSDIA	851.3934	851.3936	0.2	219.4
**b9**	GFYPSDIAV	950.4618	950.4627	0.9	303.9
**b11**	GFYPSDIAVEW	1265.5837	1265.5684	-12.1	692.2
**y2**	SK	234.1448	234.145	0.5	286.3
**y3**	YSK	397.2082	397.2083	0.2	460.9
**y4**	LYSK	510.2922	510.2929	1.2	207.4
**y5**	FLYSK	657.3606	657.3597	-1.4	207.2
**y8**	GSFFLYSK	948.4825	948.4796	-3.1	246.1
**y11**	DSDGSFFLYSK	1265.5685	1265.5684	-0.1	692.2
**y15**	PPVLDSDGSFFLYSK	1671.8265	1671.823	-2.1	254.8
**y15**	PPVLDSDGSFFLYSK	836.4169	836.4171	0.3	1488.1
**y23**	PENNYKTTPPVLDSDGSFFLYSK	1310.1343	1310.1363	1.6	1438.5
**y30**	EWESNGQPENNYKTTPPVLDSDGSFFLYSK	1150.5318	1150.5443	10.8	153.7
**y15-H2O**	PPVLDSDGSFFLYSK	827.4116	827.4062	-6.6	189.5
**Y**		136.0757	136.0756	-0.8	1002.1

Table S12. The fragment ions of corresponding peptide with LC-N30 deamidation in P1.

Ion	Sequence	m/z (prod.)	m/z	Diff (Bio, ppm)	Abund
**b2**	AS	159.0764	159.0763	-0.8	858.5
**b7**	ASQDVNT	717.305	717.3061	1.5	340
**b8**	ASQDVNTA	788.3421	788.3436	1.9	622.6
**y2**	PK	244.1656	244.1659	1.4	289.6
**y6**	PGKAPK	597.3719	597.3709	-1.7	353.1
**y10**	YQQKPGKAPK	572.8273	572.8266	-1.2	2607
**y11**	WYQQKPGKAPK	665.867	665.8671	0.1	3939.6

**y11**	WYQQKPGKAPK	444.2471	444.2465	-1.2	301.3
**y12**	AWYQQKPGKAPK	701.3855	701.3858	0.3	6360.8
**y12**	AWYQQKPGKAPK	467.9261	467.927	2	1024
**y13**	VAWYQQKPGKAPK	750.9197	750.9209	1.6	2600.8
**y14**	AVAWYQQKPGKAPK	786.4383	786.4385	0.3	583.6
**y16**	NTAVAWYQQKPGKAPK	894.4756	894.4733	-2.6	964.4
**y19**	QDVNTAVAWYQQKPGKAPK	710.7041	710.6983	-8.2	1042.7
**b7-H2O**	ASQDVNT	699.2944	699.294	-0.5	341.5
**b8-H2O**	ASQDVNTA	770.3315	770.3303	-1.6	742.3
**b8-H2O**	ASQDVNTA	385.6694	385.6709	3.9	466.2
**y19-NH3**	QDVNTAVAWYQQKPGKAPK	705.0286	705.0327	5.7	670.9
**Q**		101.0709	101.0706	-3	942.8
**W**		159.0917	159.0904	-7.7	837.3

Table S13. The fragment ions of corresponding peptide without LC-N30 deamidation in P1.

**Ion**	**Sequence**	**m/z (prod.)**	**m/z**	**Diff (Bio, ppm)**	**Abund**
**b2**	AS	159.0764	159.0766	0.9	1057.5
**b5**	ASQDV	501.2304	501.2306	0.4	410.3
**b7**	ASQDVNT	716.321	716.3226	2.3	728.2
**b7**	ASQDVNT	358.6641	358.6648	1.8	599.5
**b8**	ASQDVNTA	787.3581	787.3592	1.4	584.7
**y2**	PK	244.1656	244.1647	-3.4	522.9
**y3**	APK	315.2027	315.2035	2.6	401.4
**y6**	PGKAPK	597.3719	597.3711	-1.4	532.3
**y10**	YQQKPGKAPK	572.8273	572.8254	-3.2	2652.7
**y11**	WYQQKPGKAPK	665.867	665.8676	1	3866.3
**y12**	AWYQQKPGKAPK	701.3855	701.3865	1.4	6893
**y13**	VAWYQQKPGKAPK	750.9197	750.921	1.6	8689.3
**y15**	TAVAWYQQKPGKAPK	836.9621	836.9612	-1.1	377.8
**b7-H2O**	ASQDVNT	698.3104	698.3119	2.1	1012.8
**b8-H2O**	ASQDVNTA	769.3475	769.3481	0.8	753.1
**b8-H2O**	ASQDVNTA	385.1774	385.1774	0.1	1081.7
**y18-NH3**	DVNTAVAWYQQKPGKAPK	662.0144	662.0186	6.4	711
**y19-NH3**	QDVNTAVAWYQQKPGKAPK	704.7006	704.7069	8.9	1020.5
**Q**		101.0709	101.0705	-4.1	1044.1
**W**		159.0917	159.0904	-8.2	833.3

Table S14. The fragment ions of corresponding peptide with HC-M255 oxidation in P4.

Ion	Sequence	m/z (prod.)	m/z	Diff (Bio, ppm)	Abund
**b2**	DT	217.0819	217.0813	-2.7	1705.1
**b3**	DTL	330.166	330.1654	-1.7	397.6
**b4**	DTLM	477.2014	477.202	1.3	546.4
**y1**	R	175.119	175.1191	0.7	388.4
**y2**	SR	262.151	262.151	-0.1	1446.6
**y3**	ISR	375.235	375.2353	0.7	2799.2
**y4**	MISR	522.2704	522.2709	0.8	4043.2
**y5**	LMISR	635.3545	635.3546	0.1	3853.1
**y5**	LMISR	318.1809	318.181	0.3	1859.1
**b2-H2O**	DT	199.0713	199.0712	-0.6	528.4
**b3-H2O**	DTL	312.1554	312.1548	-1.9	1560.3
**b4-H2O**	DTLM	459.1908	459.1909	0.2	324.9
**b6-H2O**	DTLMIS	330.1571	330.1654	25.2	397.6

Table S15. The fragment ions of corresponding peptide without HC-M255 oxidation in P4.

Ion	Sequence	m/z (prod.)	m/z	Diff (Bio, ppm)	Abund
**b2**	DT	217.0819	217.0819	-0.1	17891
**b3**	DTL	330.166	330.1659	-0.1	5845.5
**y1**	R	175.119	175.1184	-3.4	5076.6
**y2**	SR	262.151	262.1514	1.6	8231.7
**y3**	ISR	375.235	375.2353	0.8	34141.5
**y4**	MISR	506.2755	506.2765	2	57825.3
**y5**	LMISR	619.3596	619.3603	1.1	45645.9
**b2-H2O**	DT	199.0713	199.0711	-1.2	6173
**b3-H2O**	DTL	312.1554	312.1553	-0.4	19091.4
**y2-NH3**	SR	245.1244	245.1293	20	4528.3
**y4-NH3**	MISR	245.1281	245.1293	4.9	4528.3

Table S16. The fragment ions of corresponding peptide with HC-M431 oxidation in P4.

Ion	Sequence	m/z (prod.)	m/z	Diff (Bio, ppm)	Abund
**b2**	WQ	315.1452	315.1437	-4.7	245.2
**b3**	WQQ	443.2037	443.204	0.5	259
**b5**	WQQGN	614.2681	614.2687	1	1287.3
**b6**	WQQGNV	713.3366	713.3358	-1.1	670.7
**y1**	K	147.1128	147.1117	-7.7	352.4
**y14**	SVMHEALHNHYTQK	855.9045	855.9077	3.8	464.7
**y14**	SVMHEALHNHYTQK	570.9387	570.9393	1	220.1
**y15**	CSVMHEALHNHYTQK	624.2823	624.2843	3.3	907.4
**y16**	SCSVMHEALHNHYTQK	653.293	653.2927	-0.4	4150.8
**y17**	FSCSVMHEALHNHYTQK	702.3158	702.314	-2.6	2461.4
**y17**	FSCSVMHEALHNHYTQK	526.9886	526.9907	3.8	500.8
**b6-NH3**	WQQGNV	348.6586	348.6591	1.2	264.6
**b16-H2O**	WQQGNVFSCSVMHEAL	624.9434	624.9506	11.5	492.4
**y16-H2O**	SCSVMHEALHNHYTQK	647.2894	647.2897	0.4	709.5
**y17-H2O**	FSCSVMHEALHNHYTQK	696.3122	696.3097	-3.7	776.9
**W**		159.0917	159.09	-10.2	323.5

Table S17. The fragment ions of corresponding peptide without HC-M431 oxidation in P4.

Ion	Sequence	m/z (prod.)	m/z	Diff (Bio, ppm)	Abund
**b2**	WQ	315.1452	315.1446	-1.7	1091
**b5**	WQQGN	614.2681	614.2682	0.2	4256.6
**b6**	WQQGNV	713.3366	713.3361	-0.6	3532.7
**y1**	K	147.1128	147.1127	-0.6	726.3
**y12**	MHEALHNHYTQK	754.8568	754.8553	-1.9	881.1
**y14**	SVMHEALHNHYTQK	847.907	847.9074	0.5	2845.5
**y14**	SVMHEALHNHYTQK	565.6071	565.6073	0.3	607.3
**y15**	CSVMHEALHNHYTQK	618.9507	618.9515	1.4	3362.8
**y16**	SCSVMHEALHNHYTQK	647.9613	647.9621	1.1	20311
**y17**	FSCSVMHEALHNHYTQK	696.9841	696.984	-0.1	11516.4
**y17**	FSCSVMHEALHNHYTQK	522.9899	522.9898	-0.2	2318.5
**b1-NH3**	W	170.06	170.0596	-2.6	597.3
**b5-NH3**	WQQGN	597.2416	597.2406	-1.7	1072.8
**b6-NH3**	WQQGNV	696.31	696.3095	-0.7	1071.4
**y17-NH3**	FSCSVMHEALHNHYTQK	691.3086	691.3152	9.5	620.3
**W**		159.0917	159.0911	-3.8	918.9
**H**		110.0713	110.0707	-5.3	617.3

Table S18. The fragment ions of corresponding peptide with HC C-terminal K loss in P5.

Ion	Sequence	m/z (prod.)	m/z	Diff (Bio, ppm)	Abund
**b2**	SL	201.1234	201.1233	-0.6	4583.9
**b3**	SLS	288.1554	288.1557	1.2	6245.4
**b4**	SLSL	401.2395	401.2395	0.1	655.8
**b5**	SLSLS	488.2715	488.2712	-0.6	2749.6
**y2**	PG	173.0921	173.092	-0.1	8476.3
**y3**	SPG	260.1241	260.1241	-0.1	4462.3
**b3-H2O**	SLS	270.1448	270.145	0.8	6343.3
**b4-H2O**	SLSL	383.2289	383.2293	1	2429.3
**b5-H2O**	SLSLS	470.2609	470.2615	1.3	2335.8

Table S19. The fragment ions of corresponding peptide without HC C-terminal K loss in P5.

Ion	Sequence	m/z (prod.)	m/z	Diff (Bio, ppm)	Abund
**b2**	SL	201.1234	201.1236	0.9	10518.6
**b3**	SLS	288.1554	288.1557	0.9	1448.4
**y1**	K	147.1128	147.1128	-0.1	931.6
**y2**	GK	204.1343	204.1344	0.6	1613
**y3**	PGK	301.187	301.1875	1.6	9557.1
**y4**	SPGK	388.2191	388.2191	0	5311.5
**y5**	LSPGK	501.3031	501.3034	0.5	2227.2
**y6**	SLSPGK	588.3352	588.3361	1.7	29379.6
**y7**	LSLSPGK	701.4192	701.4192	0	333.9
**y7**	LSLSPGK	351.2132	351.2139	2	352
**b3-H2O**	SLS	270.1448	270.1447	-0.6	1016.3
**b4-H2O**	SLSL	383.2289	383.2289	0	319.4
**y1-H2O**	K	129.1022	129.1022	-0.3	751.3
**y1-NH3**	K	130.0863	130.0857	-4.3	254.2
**y3-H2O**	PGK	283.1765	283.1765	0.1	523
**y4-H2O**	SPGK	370.2085	370.2086	0.3	259.4
**y5-H2O**	LSPGK	483.2926	483.2932	1.3	262.9
**y6-H2O**	SLSPGK	570.3246	570.3247	0.2	839.3
**y6-H2O**	SLSPGK	285.6659	285.6663	1.3	1130.7
